# Efficient motion estimation and discrete cosine transform implementation using the graphics processing units

**DOI:** 10.1371/journal.pone.0307217

**Published:** 2024-08-28

**Authors:** Shahrukh Agha, Farmanullah Jan, Haroon Ahmed Khan, Muhammad Kaleem, Mansoor Khan

**Affiliations:** 1 Department of Electrical and Computer Engineering, COMSATS University Islamabad, Islamabad, Pakistan; 2 Department of Computer Science, College of Computer Science and Information Technology, Imam Abdulrahman Bin Faisal University, Dammam, Saudi Arabia; Bocconi University: Universita Bocconi, ITALY

## Abstract

Motion Estimation (ME) and the two-dimensional (2D) discrete cosine transform (2D-DCT) are both computationally expensive parts of HEVC standard, therefore real-time performance of the HEVC may not be free from glitches. To address this issue, this study deploys the graphics processing units (GPUs) to perform the ME and 2D-DCT tasks. In this concern, authors probed into four levels of parallelism (i.e., frame, macroblock, search area, and sum of the absolute difference (SAD) levels) existing in ME. For comparative analysis, authors involved full search (FS), test zone search (TZS) of HEVC, and hierarchical diamond search (EHDS) ME algorithms. Similarly, two levels of parallelism (i.e., macroblock and sub-macroblock) are also explored in 2D-DCT. Notably, the least computationally complex multithreaded Loeffler DCT algorithm is utilized for computing 2D-DCT. Experimental results show that ME processing task corresponding to 25 frames, with each frame of size (3840×2160) pixels, is accomplished in 0.15 seconds on the NVIDIA GeForce GTX 1080, whereas the 2D-DCT task along with the image reconstruction and differencing corresponding to 25 frames took 0.1 seconds. Collectively, both ME and 2D-DCT tasks are processed in 0.25 seconds, which still leaves enough room for the encoder’s remaining parts to be executed within one second. Due to this enhancement, the resultant encoder can safely be used in real-time applications.

## Introduction

**Fig 1:**
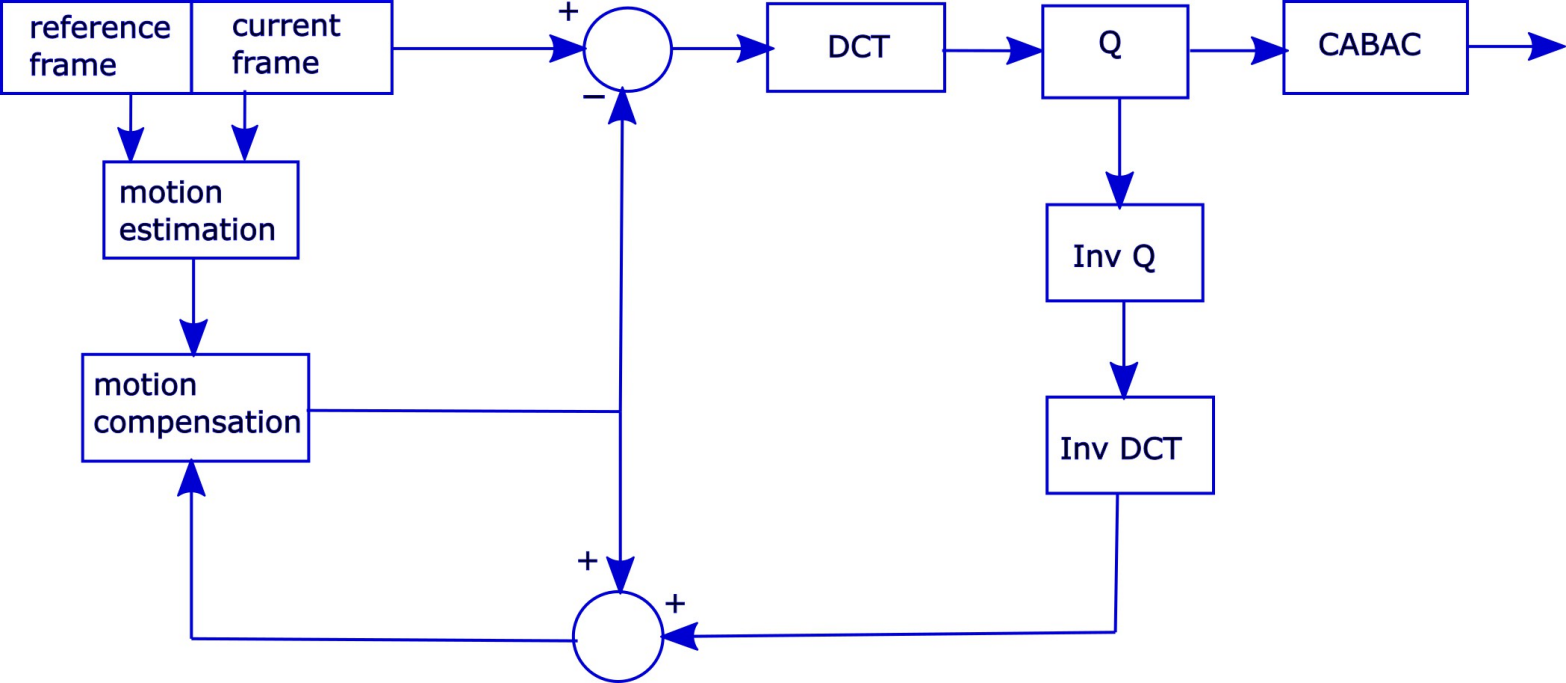
Block diagram of HEVC Encoding process.

The use of efficient algorithms for the *high efficiency video coding* (HEVC) standard to achieve high compression has significantly increased computational time of the resultant encoder [1–3]. The HEVC comprises the ME, motion compensation, 2D-DCT, inverse 2D-DCT, quantization, inverse quantization, and context adaptive binary arithmetic coding (CABAC). Among these units, both ME and 2D-DCT are considered computationally expensive [4–7]. Fig 1 shows the basic block diagram of HEVC encoding process [1–5], where “Q” is the quantization process, “Inv Q” is the inverse quantization process and “Inv DCT” is the inverse 2D-DCT process.

The ME exploits the temporal redundancy across the input images of a sequence to achieve compression. In this process, a part or the macroblock in the current frame is searched in a region (search area) in the earlier frame to find the best match of current macroblock. The criteria used for best match is known as sum of the absolute difference (SAD) metric defined as,

$$SAD(p,q)=\sum_{i=1}^{16}\sum_{j=1}^{16}\left|curr(m+i,n+j)-ref(m+p+i,n+q+j)\right|,$$ (1)

where, the parameters: *curr* and *ref* represent the current and reference frames, respectively.

The *m* and *n* are coordinates of the current frame macroblock; and *p* and *q* are coordinates of the reference frame macroblock compared to current frame macroblock. The size of macroblock is (16×16) pixels [8, 9]. In HEVC, sizes of macroblocks can be (*MB*_*SIZE*×*MB*_*SIZE*), where the *MB*_*SIZE ϵ* [8, 16, 32, 64]. In this work, value of *MB*_*SIZE* is chosen to be 16, because small macroblocks capture motion of small objects more efficiently leading to better compression. The motion vector (MV) is defined as location of the best macroblock in the search area with minimum SAD and is defined as

$$[{MV}_{x},{MV}_{y}]=\arg\;\min\;SAD(p,q),$$ (2)

where *arg* in (2) stands for the arguments, i.e., arguments of minimum SAD, which are coordinates (*p*, *q*) of the minimum SAD [8]. On the other hand, the process of ME is based on the rate distortion optimization in HEVC. It consists of the minimization of following cost metric J [8], which is defined as

$$J(p,q)=SAD(p,q)+\lambda R(MV-{MV}_{P}).$$ (3)

$$[{MV}_{x},{MV}_{y}]=\arg\;\min\;J(p,q).$$ (4)

The second term in Eq (3) is the cost of MV obtained, where the parameter λ is the Lagrange multiplier whose value depends upon the chosen quantization parameter. The *R*(*MV*−*MV*_*P*_) is the number of bits required to encode MV difference (*MV*−*MV*_*P*_) and *MV*_*P*_ is the predicted MV. Similarly, DCT [7, 10] is normally used in removing the spatial redundancy and can be considered as real part of the discrete Fourier transform (DFT). Mathematically 2D-DCT is computed as follows:

$$X(p,q)=\sum_{m=0}^{M-1}\sum_{n=0}^{N-1}a(p)a(q)x(m,n)\cos\left[\frac{\pi p(2m+1)}{2M}\right]\cos\left[\frac{\pi q(2n+1)}{2N}\right];$$ (5)

Here, 0≤*p*≤*M*−1; 0≤*q*≤*N*−1;

$$a(p)=\left\{ \begin{array}{c} 1\;if\;p=0\\ \sqrt{2}\;if\;p\neq 0\; \end{array};\right.\;a(q)=\left\{ \begin{array}{c} 1\;if\;q=0\\ \sqrt{2}\;if\;q\neq 0\; \end{array}\right..$$

In this case, *M* is taken equal to *N*. The parameters *m* and *n* are the spatial coordinates, while *p* and *q* represent the discrete frequencies. The 2D-DCT can be implemented as transform of the transform using separable property, because this technique results in reduced computational complexity. It implies that the 1D-DCT is first applied along the rows of a 2D data-matrix (*E*) and then 1D-DCT is applied along the columns of resulting matrix. Mathematically, it is computed as follows:

$$X=CEC^{T},$$ (6)

where *C* is a matrix of the DCT cosine values and *C*^*T*^ is transpose of *C*.

Exploiting inherent parallelism in a target application may potentially result in reducing the overall computational time. The single-core CPUs supporting multithreading and caches can reduce execution time of an application up to some extent thereby running multiple threads in parallel. However, when the real time constraint on an application is strict, then one must resort to more robust and resilient schemes. Literature reveals different methodologies are utilized to boost up performance of the ME/2D-DCT tasks at the algorithmic and architectural levels [8, 11, 12]. At the algorithmic level, designers often try reducing computational complexity of the relevant algorithm [6, 8, 10], but for architectural level, designers generally implement the parallel/pipelined dedicated VLSI architectures, such as the ASICs, SoC, and/or FPGAs [8, 11, 12].

In addition, the relevant research community has also utilized the *single instruction multiple data* (SIMD) vector coprocessors and multiprocessing in this concern [9, 13]. Several fast ME algorithms have been devised to alleviate computational complexity of the ME units in contemporary video encoders [6, 8]. Similarly, fast DCT computational algorithms having reduced number of additions and multiplications have also been developed [10, 11]. Besides, utilization of the *graphics processing units* (GPUs) to accelerate the video encoders’ speed is also now a hot research arena [14]. Instead of dedicating transistors to implementing large caches and efficient control flow units for CPUs, more transistors are dedicated for data processing units in GPU. Due to this reason, GPUs can efficiently execute large data in parallel. Compared with generic type CPUs, GPU is surely the best choice when accelerating a computationally complex application is needed. It is because CPUs perform sequential tasks quickly and the GPUs use parallel processing while computing tasks simultaneously with greater efficiency and speed.

In general, a typical GPU consists of the highly threaded *streaming multiprocessors* (SMs). Each SM consists of many *streaming processors* (SPs), arithmetic units (fully pipelined integer units and single precision/double precision floating point units), control logic, and instruction cache shared by SPs [14, 15]. Currently, each GPU has a graphic double data rate (GDDR) DRAM known as the global memory, which is available off-chip. In addition, a SM has a shared memory, an L1 cache, a large capacity register-file, warp scheduler(s), instruction dispatch unit(s), special function units (e.g., sine, cosine, log, and exp), load/store units shared by SPs. Shared memory and register file are partitioned among many threads. There are also memory controllers and an L2 cache, which is off-chip and shared by all SMs [14]. Though there exists a local memory, which is based on the global memory: L1 and L2 caches. The Nividia GeForce GTX 1080 GPU (based on Pascal architecture and implemented in 16 nm FinFET process) have SPs capable to run @1.6 GHz. It can provide overall throughput of 8873 Giga floating point operations per second (GFLOPs) [14]. The high-speed DRAM has 256-bit interface and can run at a data rate of 10 Gbps.

A thread is basically a program or a part of the program. In addition, a thread block consists of multiple threads oriented both horizontally and vertically. Similarly, a grid of thread blocks consists of multiple thread blocks oriented both horizontally and vertically. An SM can execute multiple threads or thread blocks concurrently depending upon the available resources [14, 16–22]. All threads inside a thread block can cooperate with each other through a *barrier synchronization mechanism*. They also share data using the shared memory. It is also found that threads belonging to different blocks cannot communicate with each other. All threads, whether belonging to the same or different block can share the global memory. At the hardware level, threads of a block are divided into groups of threads, where each group consists of 32 consecutive threads known as *warp*. All threads of a warp execute the same instruction at the same time in a *single instruction multiple thread* (SIMT) manner.

There is a fast context switching between warps. Depending upon resources, such as amount of the shared memory and register file etc., there are certain number of warps that can reside inside an SM simultaneously. However, there must be enough warps to achieve thread occupancy and hide memory latency [14, 16–22]. If consecutive threads access consecutive locations of global memory, then it leads to a coalesced memory access. In addition, the instruction level parallelism is also supported by SMs. No doubt, all these highlighted factors lead to high throughput if implemented [14, 16–22].

Moreover, it is also worth mentioning that the NVIDIA Corporation has also developed a parallel computing and programming platform known as the *Compute Unified Device Architecture* (CUDA) [23, 24]. It is a parallel computing platform and application programming interface (API), which allows software to use certain types of the GPUs for general purpose processing, i.e., an approach called general-purpose computing on GPUs (GPGPU). The researchers and developers can easily port their sequential program to GPUs using the CUDA parallel constructs for acceleration [14, 16–22]. After highlighting GPU architecture, the following text reviews contemporary work on the video encoders using GPUs.

### Literature review

Authors in [16] proposed the CUDA’s memory optimization strategies for ME. Different memory bottlenecks are identified and removed to improve the ME performance. The performance benefits of using the shared memory versus local memory are also identified. The ME algorithm utilized is the full search (FS) ME algorithm. A speed up factor of 50 is obtained while running an application on GPUs as compared with its sequential implementation on a CPU for the HD1080p (1920×1080) pixels sequence. The hardware utilized is the Intel Core i7-3770 3.40 GHz CPU being equipped with 8 GB memory and a graphic card NVIDIA GeForce GTX480. However, it is not clear whether the implementation meets real-time constraint of 25 frames per second or not.

Authors in [17] proposed a highly parallel and scalable motion estimation algorithm, known as multilevel resolution motion estimation. It combines the advantages of local full search and down-sampling. The algorithm is implemented using CUDA and runs on the GTX 460 GPU. It is worth mentioning, this design meets the coding requirements of 25 frames per second for the (2560×1600) pixels video’s format. A significant speedup as compared to serial FS ME algorithm has been achieved.

In [18], authors proposed Parallel H.264/AVC fast rate-distortion optimized motion estimation using GPU. They compared their proposed algorithm with FS, unsymmetrical multi-hexagon (UMH) search and simplified UMH search algorithms. Three GPUs were used, i.e., NVIDIA Quadro 6000, NVIDIA Tesla C2075, and NVIDIA Geforce GTX 260 for comparison purposes. The CPU used was the Core 2 Quad E5607 clocked @ 2.27GHz. They reported a speedup factor of 22 for both 720p and 1080p on average, where speedup is defined as ratio of the time consumption on CPU to GPU. They reported that for 720p and 1080p sequences, real time constraint of 25 frames per second could not be achieved, however it is achieved for the sequences having lower resolutions.

Authors in [8, 19] presented an improved full search algorithm for ME with GPU acceleration. They reported a throughput achievement of 26 frames per second for resolution of (720×576) pixels. Though real-time constraint for ME process has been achieved, however it is also not clear whether is there any proper time margin left for other parts of encoder to be executed in real-time or not? In [20], authors proposed GPU based hierarchical motion estimation for high efficiency video coding. They mentioned with the proposed scheme 41% encoding time saving can be achieved. Compared with processing time of TZS ME in CPU, processing acceleration of proposed ME on GPU can be up to 12.7 times. They further reported 200 times acceleration as compared to serial FS ME can be achieved with proposed GPU based ME compared with full search ME on CPU. Again, the question of enough time margin for the remaining parts of encoder to be executed in real time arises.

Authors in [21] presented a fast GPU based ME algorithm for the video coding. Simplified unsymmetrical multi-hexagon search is used as ME algorithm while implementing ME on GPU. The GPU utilized was the GeForce 8800 GTS PCIe graphics card and the CPU was Intel Core 2 Quad Q9400 2.66 GHz. Tiles of different sizes consisting of a number of macroblocks are utilized to obtain different throughputs. With the increasing number of tiles, throughput is improved. For example, with 3600 tiles (best case) and with image sequence “Crew” having resolution (1280×720) pixels, the time consumption of ME on GPU is 835 ms, which corresponds to a maximum speed up factor of 3.5. Although real-time constraint is met for HD 720p sequences and significant speedup is obtained as compared with the CPU based implementation, but it is not clear whether higher resolutions are supported or other parts of encoder are also executed in real-time when higher resolutions are utilized.

Authors in [22] proposed a fast GPU based motion estimation algorithm for the H.264/AVC. A modified FS ME algorithm is proposed. Significant speedup is achieved to meet real-time constraint for HD 720p sequences using GPU. They compared their proposed algorithm with FS, unsymmetrical multi-hexagon (UMH) search, and simplified UMH search algorithms. Compared to serial FS ME algorithm, speedup is over one hundred. When compared to UMH speedup is over 5; and when compared to simplified UMH ME algorithm, the speedup factor observed is over 4. The TZS [6, 25] is ME algorithm adopted by the HEVC standard. However, the requirement of spatially adjacent motion vectors, known as prediction motion vectors, to function as the starting point of search affects parallelization of algorithm. Also, the rate distortion metric (Eq 3) requires prediction motion vectors, which also inhibits the parallelization of ME algorithm.

In [26, 27], Nvidia has implemented GPU based HEVC video encoder and decoder. The encoding process can be executed at an impressive rate of 60 frames per second with image resolution of 7680 x 4320. The GPU is based on Nvidia Ada Lovelace architecture [28]. The GPU utilized based on this architecture is Nvidia L40 (AD102) with a peak performance of 90.5 tera floating point operations per second (TFLOPS).

It is worth mentioning here that the above literature review is carried out keeping in view the different frequencies of CPUs, different peak performances of GPUs, different image resolutions should be scaled to a unique value, so that a fair comparison could be done. Also serial FS ME algorithm is taken as reference ME algorithm. This comparison is done in “Results and Discussion” section.

In context with the above-mentioned issues, it is worth mentioning that this study has rendered the following contributions:

1. *For the FS ME algorithm*, *authors explored different parallelization levels*.
2. *Authors implemented latest efficient hierarchical diamond search (EHDS) ME algorithm [6] on the NVIDIA GeForce GTX 1080 GPU. Unlike TZS ME method, this scheme does not have dependencies on spatially adjacent motion vectors, leading to full parallelization of the target algorithm. EHDS ME algorithm has reduced computational complexity compared with other ME algorithms including TZS ME algorithm. The PSNR performance of EHDS ME algorithm is not only better than TZS ME algorithm but also from many other ME algorithms. Different parallelization schemes are identified to improve the throughput of ME.*
3. *In addition to the ME*, *authors also implemented and parallelized 2D-DCT*, *motion vector compensation (reconstruction)*, *and image difference (difference of current and reconstructed frames*.
4. *Authors enhanced the multithreaded Loeffler DCT algorithm [10] to use the least number of additions and multiplications while processing the target video/image data.*
5. *Finally*, *the ME along with 2D-DCT*, *reconstruction and image difference can be safely executed in real-time (25 frames per second) for the ultra-high definition (UHD) sequences* (3840×2160) *pixels and still leaving enough time margin for other video encoder’s parts*.

## Proposed methodology

**Fig 2:**
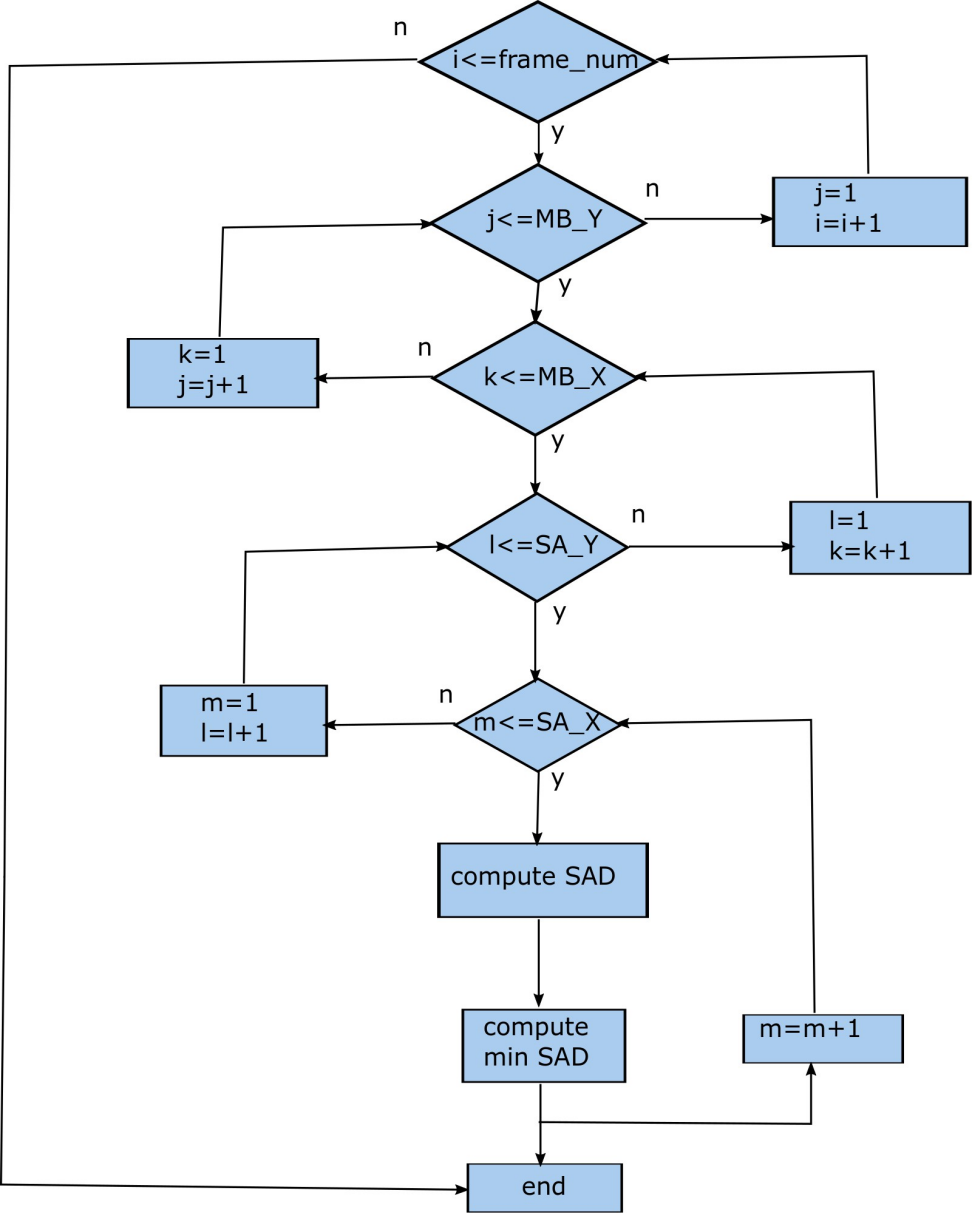
Flowchart for Algorithm 1 depicting serial FS ME process.

The flowchart for the FS ME process (Algorithm 1) can be seen in Fig 2. The parameter *frame*_*num* denotes the number of frames. The *MB*_*Y* and *MB*_*X* denote the number of vertical macroblocks and horizontal macroblocks, respectively. Similarly, the *SA*_*Y* and *SA*_*X* denote the vertical and horizontal dimension of the search area. Lastly, the *CMV* represents candidate *motion vector* (MV). There are two more inner most loops (not shown) for computing the SAD metric sequentially. This is a sequential algorithm involving billions of the arithmetic operations and memory accesses corresponding to search area range of [32 x 32] and the UHD sequences [6, 8].

**Fig 3:**
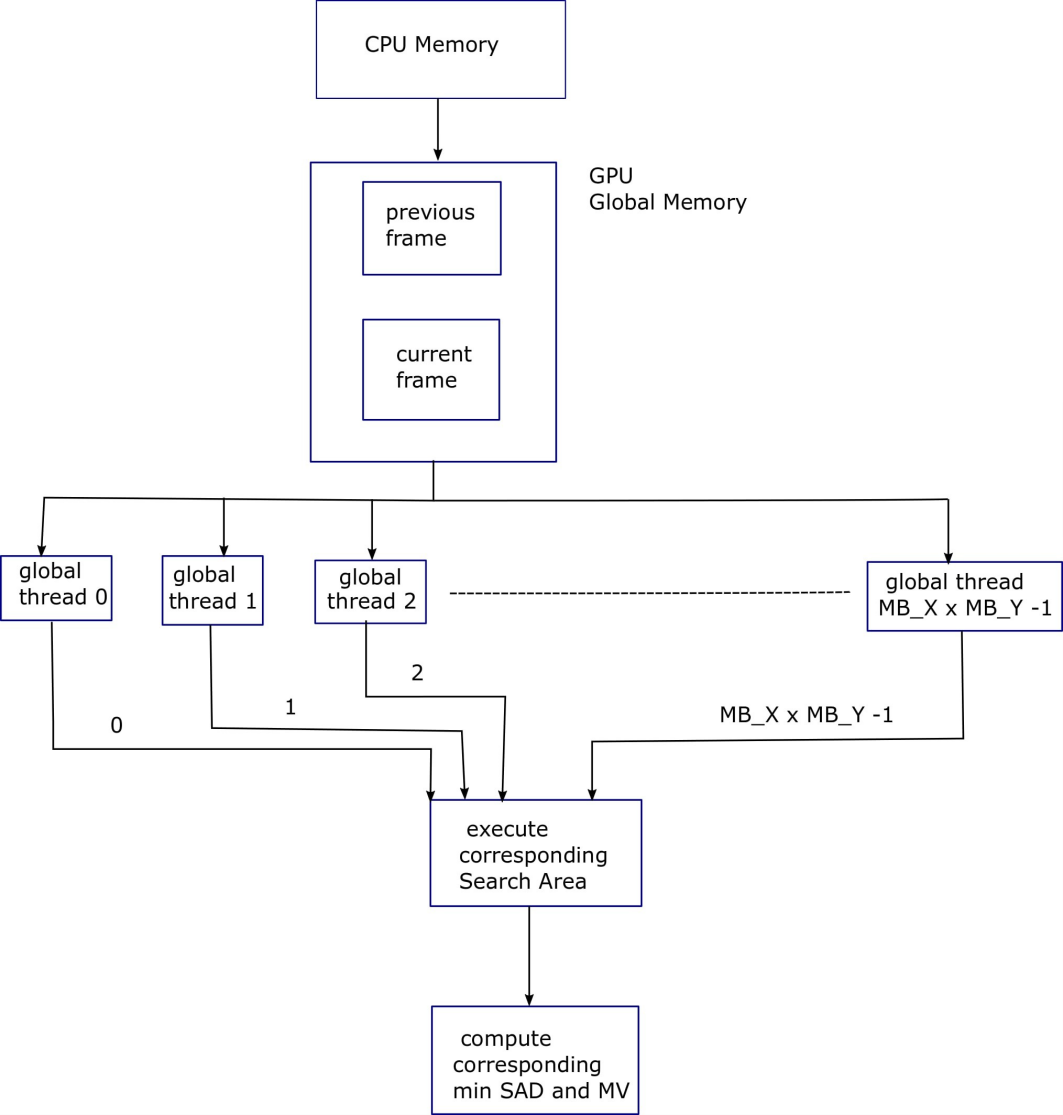
Block diagram for Algorithm 2, explaining the parallelization provided by the GPU threads for FS ME process.

Algorithm 2 shows pseudocode of FS ME algorithm in which the second and third for-loops of Algorithm 1 are parallelized. For this purpose, a 2D grid of the global threads, with dimensions (*MB*_*X*×*MB*_*Y*) is created in the CUDA C [14]. Depending upon the SMs available in GPU, these threads will be executed concurrently and will execute the following code in parallel. Fig 3 shows block diagram for Algorithm 2, explaining the parallelization provided by the GPU threads for FS ME process.

**Algorithm 2:** Pseudocode for the macroblock level parallel FS ME algorithm.

***for*** *i in 1 to frame_num loop*

 {

 • Load frames into GPU global memory.

 • 2D grid (*MB*_*X*×*MB*_*Y*) of global threads executes the following instructions simultaneously.

    ***for*** *l in* 1 *to SA_Y loop*

   ***for*** *m in* 1 *to SA_X loop*

    {

     ■ Compute SAD corresponding to *CMV*(*l*,*m*)

     ■ Compute minimum SAD and the corresponding *MV*(*l*,*m*)

    }

 }

**Fig 4:**
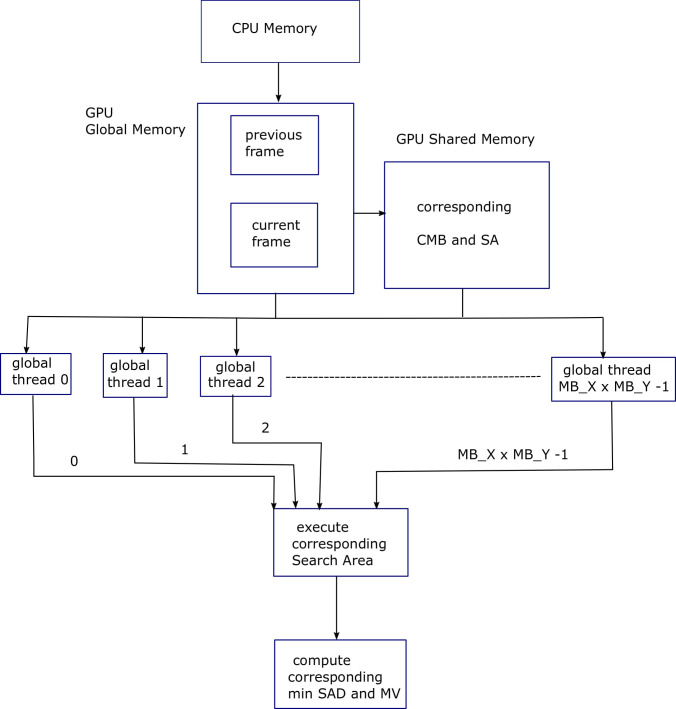
Block diagram for Algorithm 3, explaining the parallelization provided by the GPU threads for FS ME process. Macroblock level parallelization has been depicted.

The pseudocode in Algorithm 3 is same as that of Algorithm 2, except the shared memory is used to store the current macroblock (CMB) and search area (SA) for fast access and efficient data reuse. Shared memory is the fastest memory after register-file in GPU. Corresponding data is loaded into the corresponding shared memories in a coalesced manner. Fig 4 shows Block diagram for Algorithm 3, explaining the parallelization provided by the GPU threads for FS ME process. macroblock level parallelization has been depicted.

**Algorithm 3:** Pseudocode for Macroblock level parallel FS ME algorithm with shared memory.

***for*** *i in* 1 *to frame_num loop*

 {

   •Load frames into GPU global memory.

   •2D grid (*MB*_*X*×*MB*_*Y*) of global threads is executing the following instructions simultaneously.

   •Load corresponding CMB and SA in the corresponding shared memories.

 ***for*** *l in* 1 *to SA_Y loop*

    ***for*** *m in* 1 *to SA_X loop*

   {

    • Compute SAD corresponding to *CMV*(*l*,*m*).

    • Compute minimum SAD and the corresponding *MV*(*l*,*m*).

   }

 }

**Fig 5:**
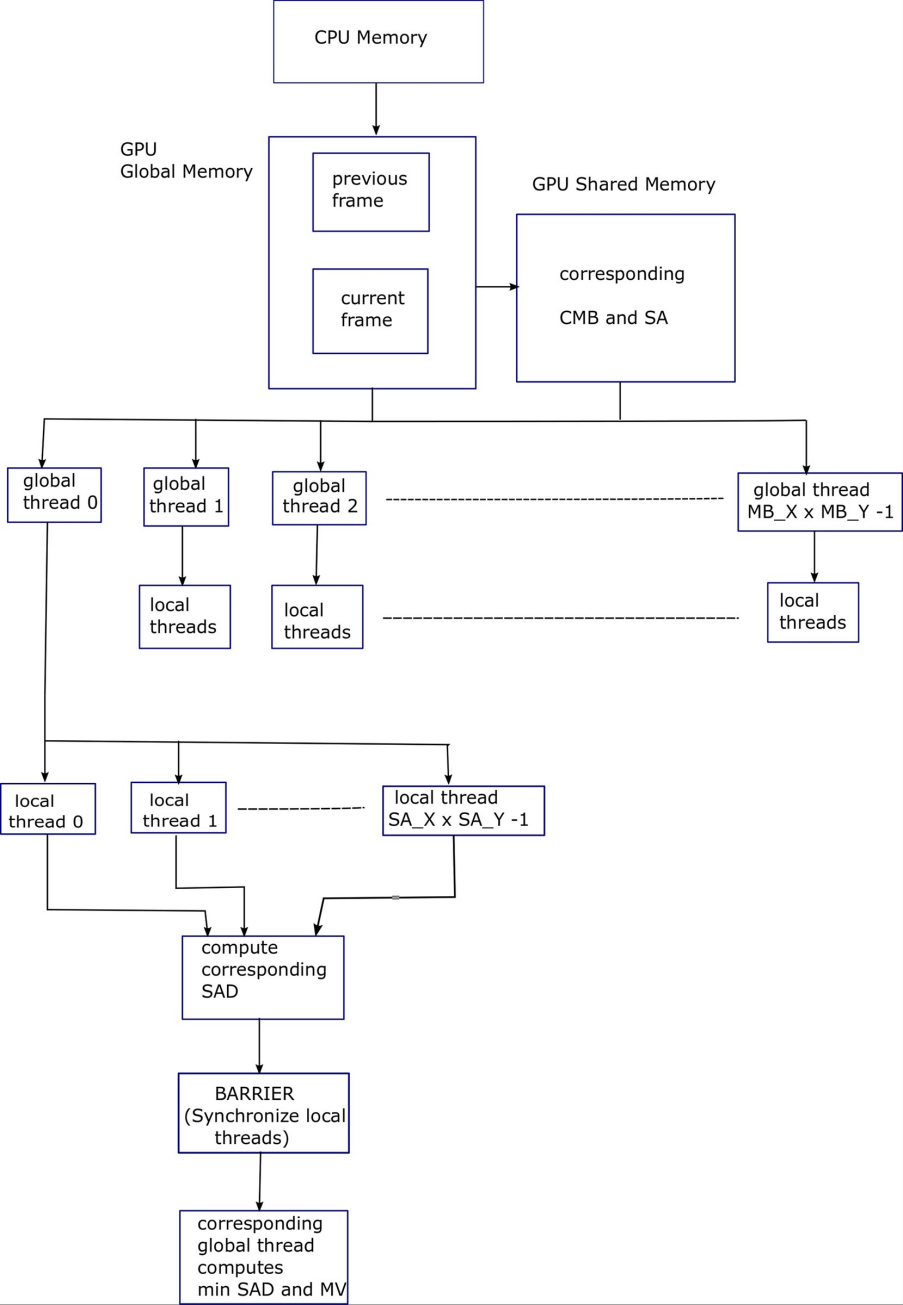
Block diagram for Algorithm 4, explaining the parallelization provided by the GPU threads for FS ME process. SA level and Macroblock level parallelization has been depicted.

Algorithm 4 shows pseudocode for the FS ME algorithm in which second, third, fourth, and fifth for-loops of Algorithm 1 are parallelized. For this purpose, a 2D grid of the global threads having dimensions (*MB*_*X*×*MB*_*Y*) pixels is created in CUDA C. Similarly, a 2D thread block of the local threads with dimensions (*SA*_*X*×*SA*_*Y*) pixels per global thread is created. Macroblock level loops (second and third loops) are parallelized using global threads while the search-area level loops (fourth and fifth loops) are parallelized using local threads. Search areas (SA) and current macroblocks (CMB) are loaded into corresponding shared memories in a coalesced manner. A barrier mechanism for synchronizing the local threads is also used. Fig 5 shows block diagram for Algorithm 4, explaining the parallelization provided by the GPU threads for FS ME process. SA level and Macroblock level parallelization has been depicted.

**Algorithm 4:** Pseudocode for search area and Macroblock level parallel FS ME algorithm.

***for*** *i in* 1 *to frame_numb loop*

 {

  • Load frames into GPU global memory.

  • 2D grid (*MB*_*X*×*MB*_*Y*) of global threads is executing following instructions simultaneously.

  • Load corresponding CMB and SA in the corresponding shared memories using four pixels per local thread for SA and one pixel per local thread for CMB.

  • 2D block (*SA*_*X*×*SA*_*Y*) of local threads executes the following instructions simultaneously.

  • Each local thread computes one SAD corresponding to one CMV.

  • Synchronize threads.

  • The corresponding main global thread executes following instructions:

    ***for*** *l in* 1 *to SA_Y loop*

      ***for*** *m in* 1 *to SA_X loop*

    • Compute minimum SAD and the corresponding *MV*(*l*,*m*).

      }

**Fig 6:**
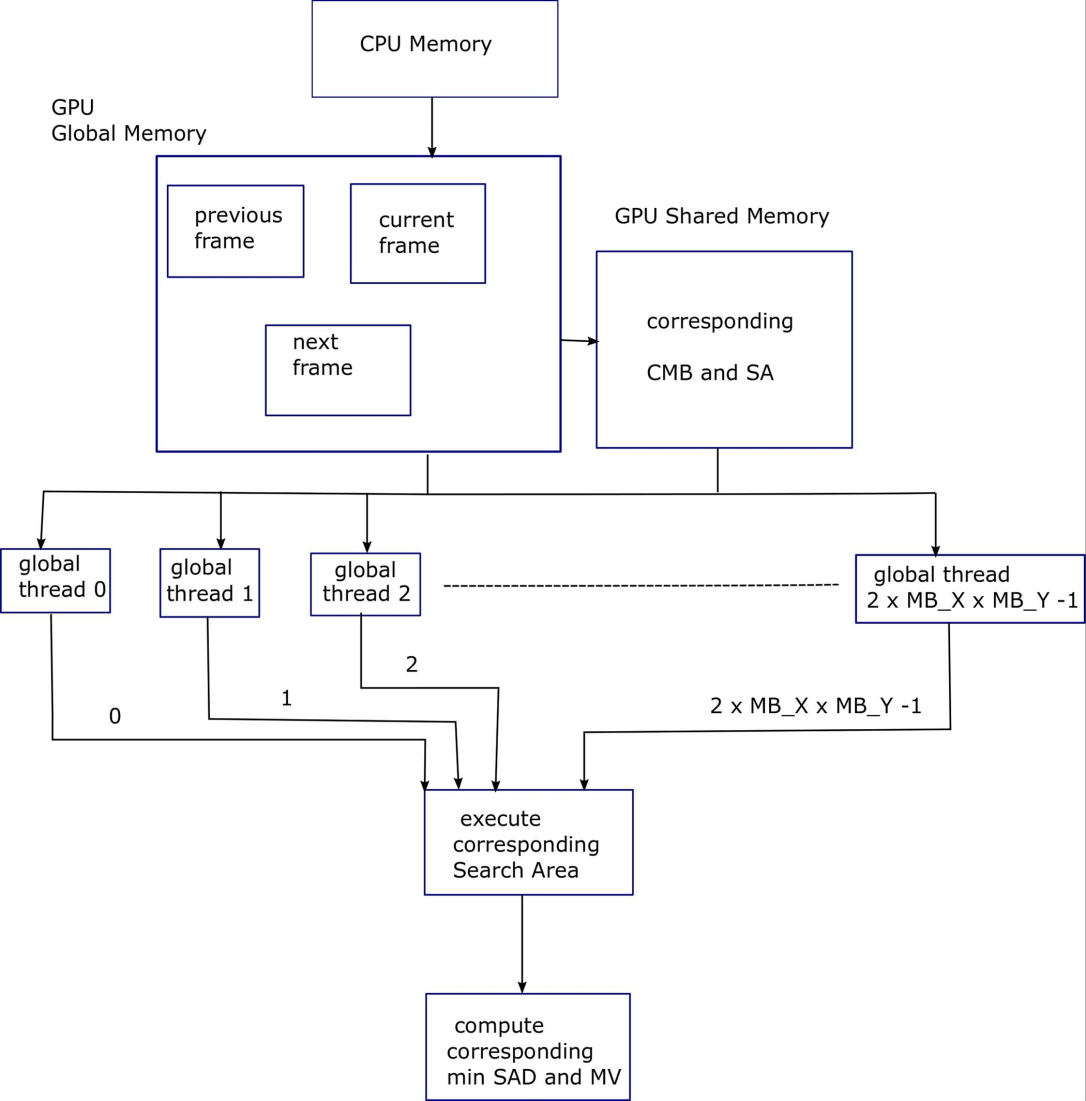
Block diagram for Algorithm 5, explaining the parallelization provided by the GPU threads for FS ME process. Frame level and macroblock level parallelization has been depicted.

Algorithm 5 shows parallelization of the frame-level loop (first loop) of the FS ME algorithm. A 2D grid of threads with dimensions (2×*MB*_*X*×*MB*_*Y*) pixels is used, i.e., ME process corresponding to 3 frames is being executed in parallel. Here 2 ME processes are in parallel. During the first iteration of frame level for-loop, 3 consecutive frames are loaded into the device global memory. Next, with each succeeding iteration, 2 consecutive frames are loaded. Similarly, when 3 ME processes are parallelized, then 4 consecutive frames in the first iteration will be loaded into device memory. With each succeeding iteration, 3 consecutive frames will be loaded and so on. Fig 6 shows block diagram for Algorithm 5, explaining the parallelization provided by the GPU threads for FS ME process. Frame level and Macroblock level parallelization has been depicted.

**Algorithm 5:** Pseudocode for parallelizing frame-level loop (1^st^ loop) of FS ME algorithm.

***for*** *i in* 1 *to frame_num/2 loop*

 {

  • Load frames into GPU global memory.

  • 2D grid (2×*MB*_*X*×*MB*_*Y*) of global threads executes following instructions simultaneously.

  • Load corresponding CMB and SA in the corresponding shared memories.

      ***for*** *l in* 1 *to SA_Y loop*

       ***for*** *m in* 1 *to SA_X loop*

                   {

   • Compute SAD corresponding to *CMV*(*l*,*m*)

   • Compute minimum SAD and the corresponding *MV*(*l*,*m*)

                    }

            }

Algorithm 6 shows pseudocode for the sequential SAD computation. As can be seen there are two for-loops. A (16×16) pixels macroblock has 256 absolute difference and addition operations. In addition, each for-loop has associated 3 instructions, i.e., condition checking, iteration increment, and *goto* instruction per iteration. So, for a (16×16) pixels macroblock, it amounts to (256×2+(16×3+3)×16) instruction executions, which equals 1328 instruction executions, where memory access instructions are excluded and though absolute difference operation is a multi-instruction operation, it is assumed here a single operation for simplicity.

**Algorithm 6:** Pseudocode for the SAD computation sequentially.

 ***for*** *l in* 1 *to MB_SIZE loop*

  ***for*** *m in* 1 *to MB*_*SIZE loop*

   • *SAD*1 = *SAD*1+*abs*(*curr*(*l*,*m*)−*ref*(*l*,*m*))

Algorithm 7 shows pseudocode for parallelization of the SAD computation by unrolling the inner for-loop and multithreading the external for-loop. Assuming *MB*_*SIZE* equals 16, block of (16×16) pixels local threads is executing the instructions shown. The *x* and *y* are the horizontal and vertical coordinates of thread block, respectively. *AD* is the absolute difference and is a 2D array mapped to shared memory. A total of 256 absolute operations are computed in parallel. Next, these threads are synchronized. Then, 16 threads compute the SADs, where each of 16 threads compute SAD corresponding to the macroblock row as shown by the shared memory array variable *SAD*1. Threads are again synchronized, and at the end, a single thread computes 16 resultant SADs. Now maximum operations per thread are reduced significantly, excluding conditional instructions for threads. The maximum operations per thread per macroblock are now approximately (1+16+16), which equals to 33. As before the memory access instructions are excluded. There is further possibility of reducing these instructions per thread, e.g., by using 32 threads instead of 16 threads for computing SAD, where (16×16) absolute differences block is broken down into four (8×8) absolute differences blocks, and each (8×8) block is being added by 8 threads. Next, threads are synchronized.

**Algorithm 7:** Pseudocode for the parallelization of the SAD metric calculation.

 • Block of (*MB*_*SIZE*×*MB_SIZE*) pixels local threads execute the following instructions simultaneously.

  • A*D*[*y*][*x*] = *abs*(*curr*[*y*][*x*]−*ref*[*y*][*x*])

 Synchronize threads.

 Following instructions are executed by MB_SIZE threads.

  • S*AD*1[*y*] = *SAD*1[*y*]+*AD*[*y*][0]

  • - - - - - - - - - - - - - - - -

  • *SAD*1[*y*] = *SAD*1[*y*]+*AD*[*y*][*MB*_*SIZE*−1]

  Synchronize threads.

The following instructions are executed by a single thread.

  ***for*** *i in* 0 *to MB_SIZE*−1 *loop*

  • *s* = *s*+*SAD*1[*i*]

 **return (*s*)**

Then, again (2×2) thread block is utilized, in which each thread adds the 8 resultant intermediate SADs out of 32 resultant intermediate SADs in parallel. Threads are synchronized again, and finally, a single thread adds 4 resultant intermediate SADs. Though in this case the maximum number of instruction executions per thread are reduced to (1+8+8+4), which equals to 21 excluding memory access and conditional instructions. However, this later case has more complex coding structure and involves more conditional instructions.

**Fig 7:**
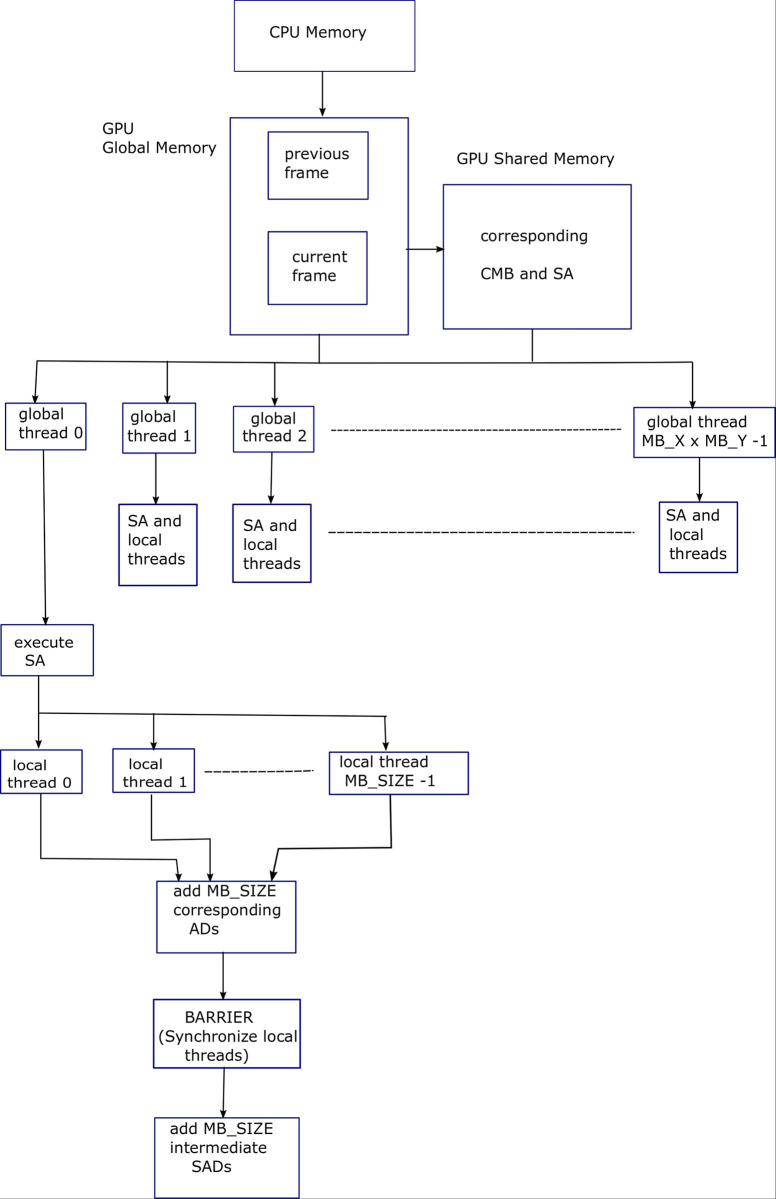
Block diagram for Algorithm 7, explaining the parallelization provided by the GPU threads for FS ME process. SAD level parallelization has been depicted.

After identifying 4 levels of parallelism in FS ME algorithm, i.e., frame level, macroblock level, search area level, and SAD level parallelization; this work now proceeds towards the parallelization of a recently proposed fast ME algorithm by the authors known as the EHDS ME algorithm [6]. The first step in EHDS ME algorithm is to produce multiple resolution images of the subject frames. Fig 7 shows block diagram for Algorithm 7, explaining the parallelization provided by the GPU threads for FS ME process. SAD level parallelization has been depicted.

Algorithm 8 shows pseudocode for producing multiple resolution (two) images of the frames sequentially. Here, the size of macroblock is *MB*_*SIZE*, which is 16 and *MB*_*SIZE*1 is 8. Resolution of (8×8) pixels is obtained from (16×16) pixels macroblock by averaging every (2×2) non-overlapping blocks. Similarly, the second resolution of (4×4) is obtained from (8×8).

**Algorithm 8:** Pseudocode for producing multiple resolution (two) images of frames, sequentially.

***for*** *y in* **1** *to MB_Y loop*

 ***for*** *x in* 1 *to MB*_*X loop*

  {

    ***for*** *i in* **1** *to MB_SIZE/*2 *loop*

    ***for*** *j in* 1 *to MB_SIZE*/2 *loop*

    • MB1 = average 2 x 2 non-overlapping squares of the macroblock.

  ***for*** *i in* 1 *to MB_SIZE*1/2 *loop*

   ***for*** *j in* 1 *to MB_SIZE*1/2 *loop*

    • MB2 = average (2×2) non-overlapping squares of the macroblock1.

     }

**Fig 8:**
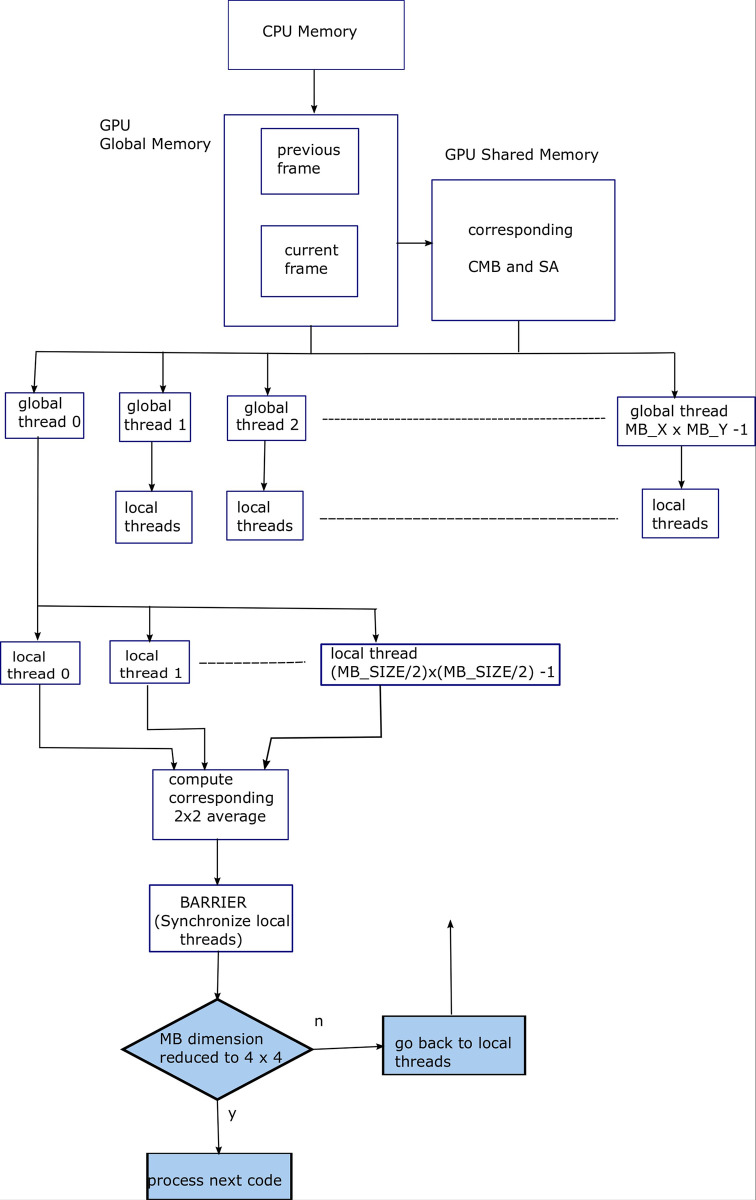
Block diagram for Algorithm 9, explaining the parallelization provided by the GPU threads for EHDS ME process. Creation of hierarchy of multiple resolution images.

Algorithm 9 shows pseudocode for parallelizing for-loops of Algorithm 8. For this purpose, a grid of global threads of dimension (*MB*_*X*×*MB*_*Y*) pixels is initiated. In addition, the third and fourth for-loops are parallelized using a block of local threads of dimensions (*MB*_*SIZE*/2×*MB*_*SIZE*/2). In this case, each thread will compute average of the non-overlapping (2×2) block of pixels. These local threads are also responsible for loading macroblock into shared memory in parallel. The threads are then synchronized. Following that, the fifth and sixth for-loops are parallelized using block of local threads of dimensions (*MB*_*SIZE*1/2×*MB*_*SIZE*1/2), where *MB*_*SIZE* is 16 and *MB*_*SIZE*1 is 8. In this case again, each thread computes the average of non-overlapping (2×2) block of pixels. Here, *x* and *y* are the horizontal and vertical coordinates of thread block, respectively. Fig 8 shows block diagram for Algorithm 9, explaining the parallelization provided by the GPU threads for EHDS ME process. Creation of hierarchy of multiple resolution images is depicted.

**Algorithm 9:** Pseudocode for parallelizing the external macroblock level for loops of Algorithm 8.

• Following instructions are being executed by a grid (*MB*_*X*×*MB*_*Y*) of global threads simultaneously.

• The following instructions are being executed by a (*MB*_*SIZE*/2×*MB*_*SIZE*/2) block of local threads.

• Load macroblock in shared memory

   MB1[y][x]=(MB[2*y][2*x]+MB[2*y][2*x+1]+MB[2*y+1][2*x]+MB[2*y+1][2*x+1])/4

• Synchronize threads.

• The following instructions are being executed by a (*MB*_*SIZE*1/2*xMB*_*SIZE*1/2) block of local threads.

   MB2[y][x]=(MB1[2*y][2*x]+MB1[2*y][2*x+1]+MB1[2*y+1][2*x]+MB1[2*y+1][2*x+1])/4

**Fig 9:**
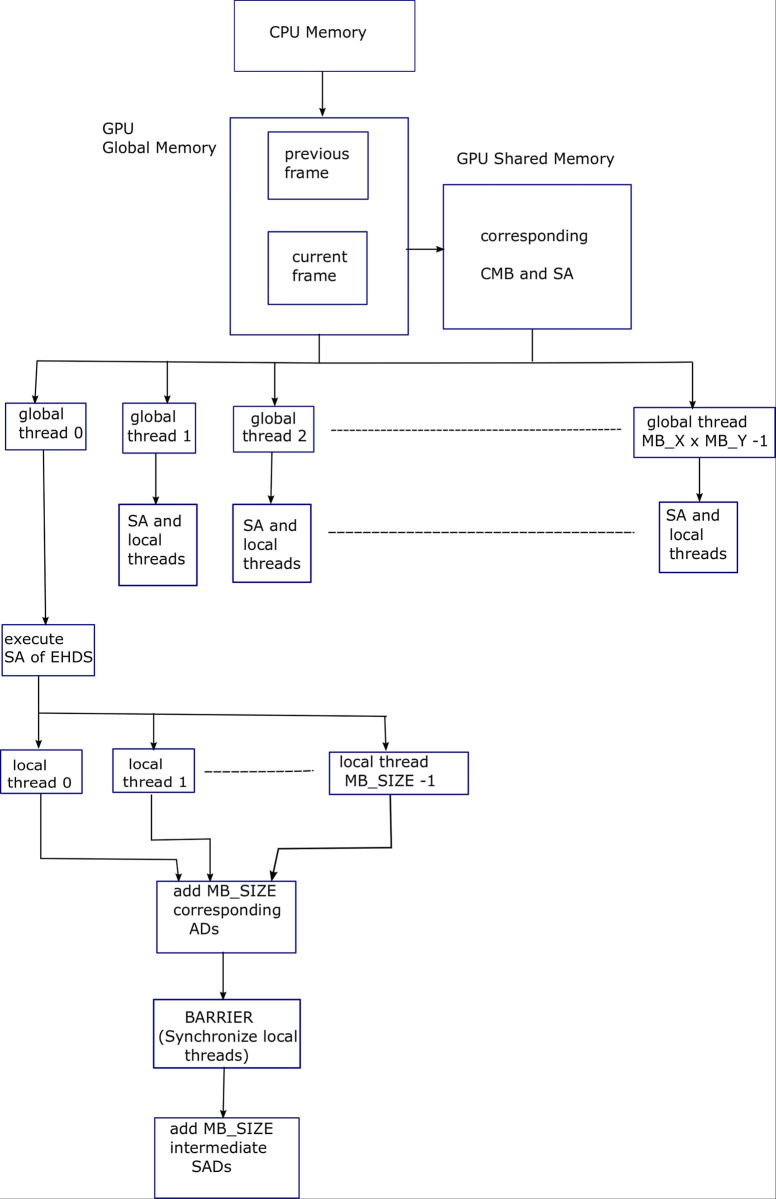
Block diagram for Algorithm 11, explaining the parallelization provided by the GPU threads for EHDS ME process. Macroblock level and SAD level parallelization is depicted.

The pseudocode given in Algorithm 10 shows serial implementation of the EHDS ME algorithm, while the pseudocode given in Algorithm 11 shows that second and third for-loops in Algorithm 10 are replaced by 2D grid of global threads of dimensions (*MB*_*X*×*MB*_*Y*) pixel. Fig 9 shows block diagram for Algorithm 11, explaining the parallelization provided by the GPU threads for EHDS ME process. Macroblock level and SAD level parallelization is depicted.

**Algorithm 10:** Pseudocode for the serial implementation of EHDS ME algorithm.

***for*** *i in* 1 *to frame*_*num loop*

  ***for*** *j in* 1 *to MB*_*Y loop*

    ***for*** *k in* 1 *to MB*_*X loop*

      {

       • Execute EHDS in a given search area.

       • Compute minimum SAD and the corresponding *MV*(*l*,*m*).

               }

**Algorithm 11:** Pseudocode for the parallel implementation of EHDS ME algorithm.

***for*** *i in* 1 *to frame*_*num loop*

{

 • The following instructions are being executed by a grid of global threads of dimension (*MB*_*X*×*MB*_*Y*)

 • The following instructions are being executed by a block of local threads of dimension (*MB*_*SIZE*×*MB*_*SIZE*)

 • Load corresponding CMB and SA in the corresponding shared memories.

               {

  • Execute EHDS in a given search area.

  • Compute minimum SAD and the corresponding *MV*(*l*,*m*).

                }

   }

The SA and CMB are loaded into corresponding shared memories in a coalesced manner by the local threads. After finding parallelism at macroblock level, second parallelism exists at the SAD level, as was the case in FS ME algorithm given in Algorithm 7. The block of local threads are computing the SAD in parallel. Though frame-level parallelization can also be utilized, but utilizing all levels of parallelization did not give any significant improvement as compared to utilizing few levels of parallelization. It is because search area parallelization cannot be used together with SAD level parallelization. Second, as the number of SMs are limited, using too many threads instead creates serialization effect and hence after certain number of threads, any further multithreading does not give significant benefits.

**Fig 10:**
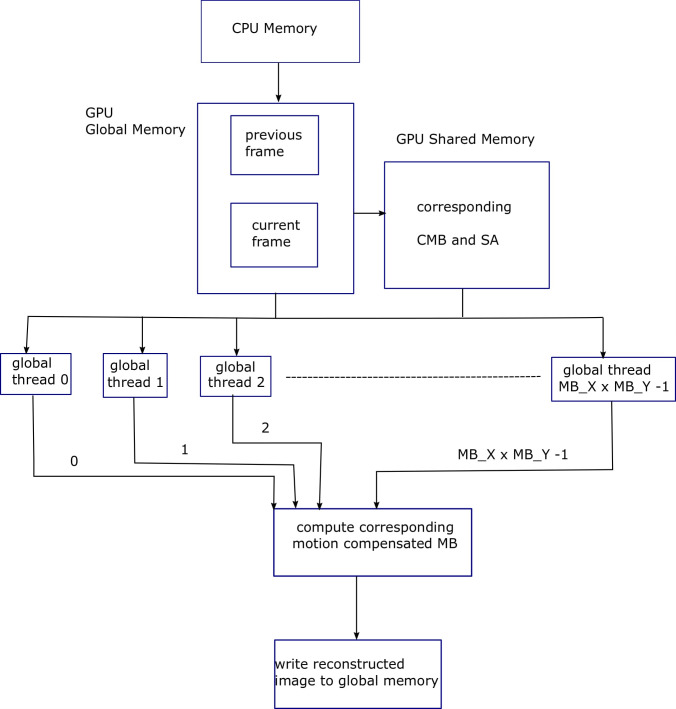
Block diagram for Algorithm 13, explaining the parallelization achieved using GPU threads, for motion compensation process.

In addition, search area CMVs in EHDS algorithm have less regularity and are not deterministic, i.e., CMV locations are dependent on the nature of images [6], hence search area level parallelization is not utilized for EHDS algorithm. Algorithm 12 shows pseudocode for serial implementation of motion compensation or reconstruction while pseudocode in Algorithm 13 shows that the first and second for-loops in Algorithm 12 are replaced by 2D grid of global threads of dimensions (*MB*_*X*×*MB*_*Y*) pixel. Corresponding motion vector is loaded into register or shared memory for fast processing and data reuse. The *previous*_*MB* is the reference (previous) frame macroblock and *mv*_*x* and *mv*_*y* are the corresponding motion vectors. Fig 10 shows block diagram for Algorithm 13, explaining the parallelization achieved using GPU threads, for motion compensation process.

**Algorithm 12:** Pseudocode for the motion compensation or reconstruction.

    ***for*** *i in* 1 *to MB*_*Y*

     ***for*** *j in* 1 *to MB*_*X*

      {

           ***for*** *x in* 1 *to MB*_*SIZE loop*

        ***for*** *y in* 1 *to MB*_*SIZE loop*

            • reconstructed_MB(x,y)=previous_MB(x+mv_x,y+mv_y)

        }

**Algorithm 13:** Pseudocode for parallel implementation of motion compensation or reconstruction.

• The following code is being executed by a grid (*MB*_*X*×*MB*_*Y*) of global threads simultaneously.

• Load motion vector into register or shared memory.

   ***for*** *x in* 1 *to MB*_*SIZE loop*

      ***for*** *y in* 1 *to MB*_*SIZE loop*

      • reconstructed_MB(x,y)=previous_MB(x+mv_x,y+mv_y)

**Fig 11:**
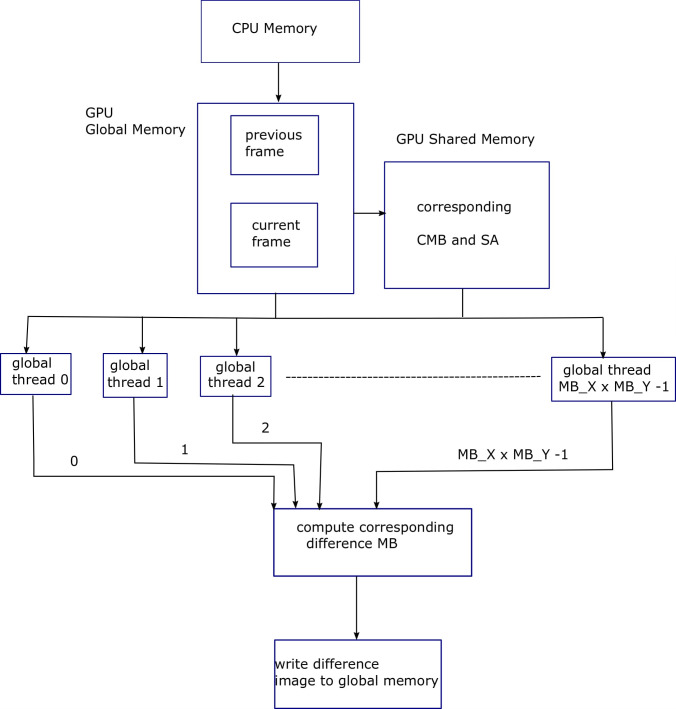
Block diagram for Algorithm 15, explaining the parallelization achieved using GPU threads, for image differencing process.

Algorithm 14 shows pseudocode for the serial implementation of image differencing, i.e., difference between current frame and its reconstruction, while Algorithm 15 shows its parallel version. The *current*_*MB* is the current frame macroblock. It is important to mention here that two for-loops shown in Algorithms 13 & 15 can further be parallelized when local threads are utilized, but the resultant improvement is not much significant. After image differencing comes the stage of 2D-DCT. As shown in Eq 6, due to the separable property of 2D-DCT [7], it can be implemented as transform of the transform. Fig 11 shows block diagram for Algorithm 15, explaining the parallelization achieved using GPU threads, for image differencing process.

**Algorithm 14:** Pseudocode for the serial implementation of the image differencing.

 ***for*** *i in* 1 *to MB*_*Y*

  ***for*** *j in* 1 *to MB*_*X*

     {

        ***for*** *x in* 1 *to MB*_*SIZE loop*

      ***for*** *y in* 1 *to MB*_*SIZE loop*

       • difference_MB(x,y)=current_MB(x,y)–reconstructed_MB(x,y)

     }

**Algorithm 15:** Pseudocode for the parallel implementation of image differencing.

• The following code is being executed by a grid (*MB*_*X*×*MB*_*Y*) of global threads simultaneously.

  ***for*** *x in* 1 *to MB*_*SIZE loop*

    ***for*** *y in* 1 *to MB*_*SIZE loop*

      • difference_MB(x,y)=current_MB(x,y)–reconstructed_MB(x,y)

Algorithm 16 shows pseudocode for the serial implementation of 2D-DCT as transform of transform. Here 1D-DCT is applied along rows of blocks. Then in the second step, 1D-DCT is applied along columns of result, leading to 2D-DCT. The number of blocks horizontally are (2×*MB*_*X*) and number of blocks vertically are (2×*MB*_*Y*), since size of block is (8×8) pixel, which is half size of macroblock in ME process.

**Algorithm 16:** Pseudocode for serial implementation of 2D-DCT as transform of transform/

  ***for*** *m in* 1 *to* 2* *MB*_*Y loop*

    ***for*** *n in* 1 *to* 2* *MB*_*X loop*

      {

        • Compute 1D-DCT

        • Compute 2D-DCT

           }

Algorithm 17 shows pseudocode for the serial implementation of conventional 1D-DCT applied along the rows of data block. Here *MB*_*SIZE*1 has a value of 8, as 2D-DCT is applied over (8×8) blocks. The *coeff* represents the 2D matrix of DCT coefficients (cosine values).

**Algorithm 17:** Pseudocode for serial implementation of conventional 1D-DCT.

 ***for*** *row in* 1 *to MB*_*SIZE loop*

  ***for*** *k in* 1 *to MB*_*SIZE*1 *loop*

    {

     • *temp* = 0

    ***for*** *k*1 *in* 1 *to MB*_*SIZE*1 *loop*

     {

     • temp=temp+mb[row][k1]*coeff[k][k1]

     }

     • *dct*_1*d*[*row*][*k*] = *temp*

 }

Algorithm 18 shows pseudocode for conventional 1D-DCT applied along the columns of 1D-DCT already computed in Algorithm 17 to compute 2D-DCT.

**Algorithm 18:** Pseudocode for serial 2D-DCT from 1D-DCT.

 ***for*** *col in* 1 *to MB*_*SIZE*1 *loop*

  ***for*** *k in* 1 *to MB*_*SIZE*1 *loop*

   {

    • *temp* = 0

   ***for*** *k*1 *in* 1 *to MB*_*SIZE*1 *loop*

  {

    • temp=temp+dct_1d[k1][col]*coeff[k][k1]

   }

    • *dct*_2*d*[*k*][*col*] = *temp*

   }

Algorithm 19 shows pseudocode for parallelization of conventional serial 2D-DCT as given in Algorithms 17 & 18. The values of *col* and *row* in Algorithm 19 are 8 each in context with size of (8×8) pixels block. The external two for-loops in Algorithms 17 & 18 are replaced by 2D thread block of local threads. The difference image block is read into shared memory in parallel in a coalesced manner, i.e., each local thread reads in a single pixel from the global memory. Similarly, DCT coefficients are read into shared memory in a coalesced manner, as there is a lot of data reuse. Then threads are synchronized. Then each local thread computes one 1D-DCT value from the block rows.

**Algorithm 19:** Pseudocode for the parallelization of the conventional serial 2D-DCT.

• 2D grid (2*xMB*_*X*×2*xMB*_*Y*) of global threads is executing the following instructions simultaneously.

• 2D block (*col*×*row*) of local threads is executing the following instructions simultaneously.

• Read corresponding difference macroblock in a shared memory in parallel.

• Read DCT coefficients in a shared memory in parallel.

• Synchronize threads.

• Each 1D DCT value is computed by a separate thread (block rows and coefficient columns)

• Synchronize threads.

• Each 1D-DCT value is computed by a separate thread (1D-DCT columns and coefficient columns).

**Fig 12:**
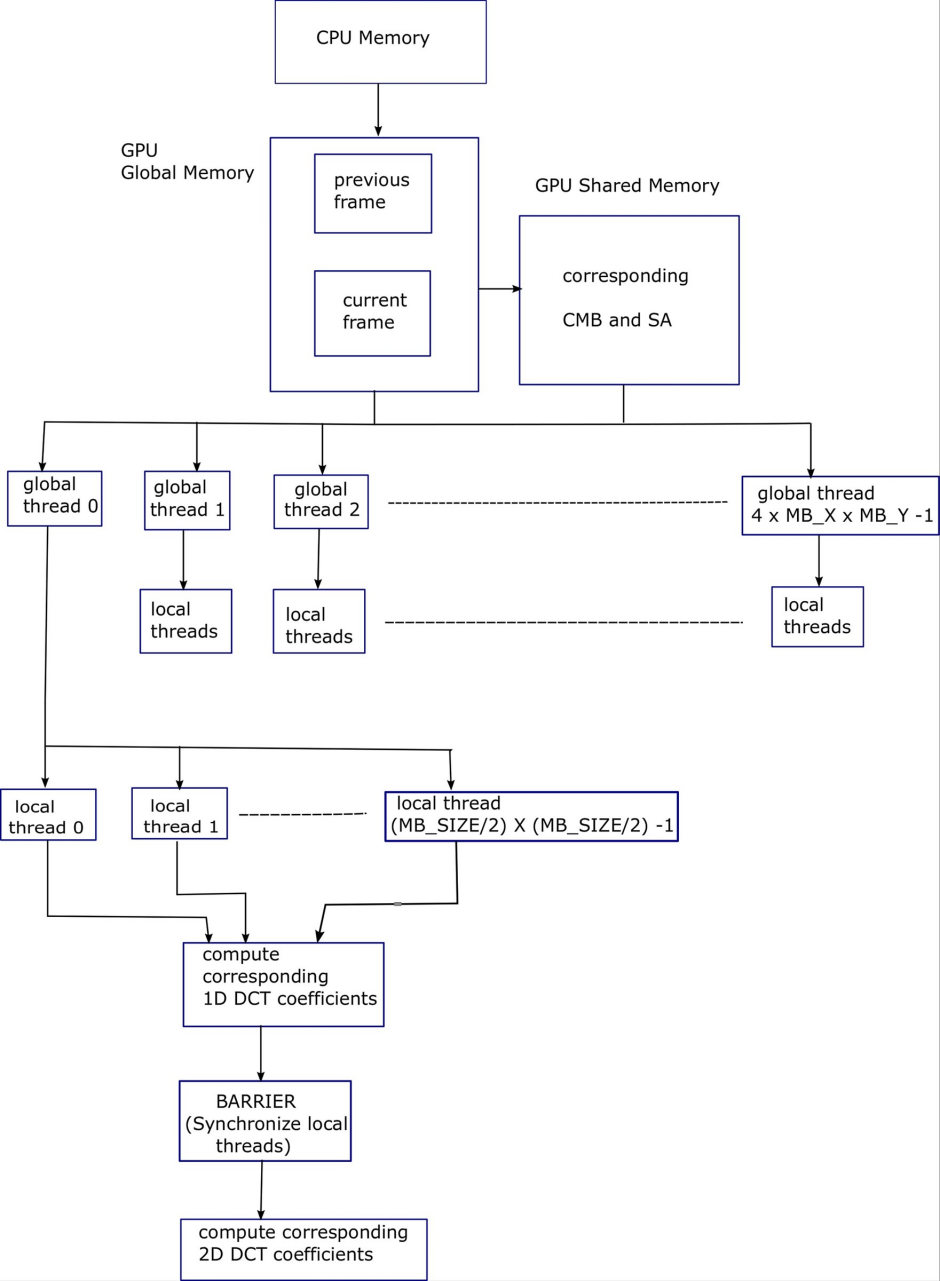
Block diagram for Algorithm 19, explaining the parallelization achieved using GPU threads, for conventional 2D DCT process.

**Fig 13:**
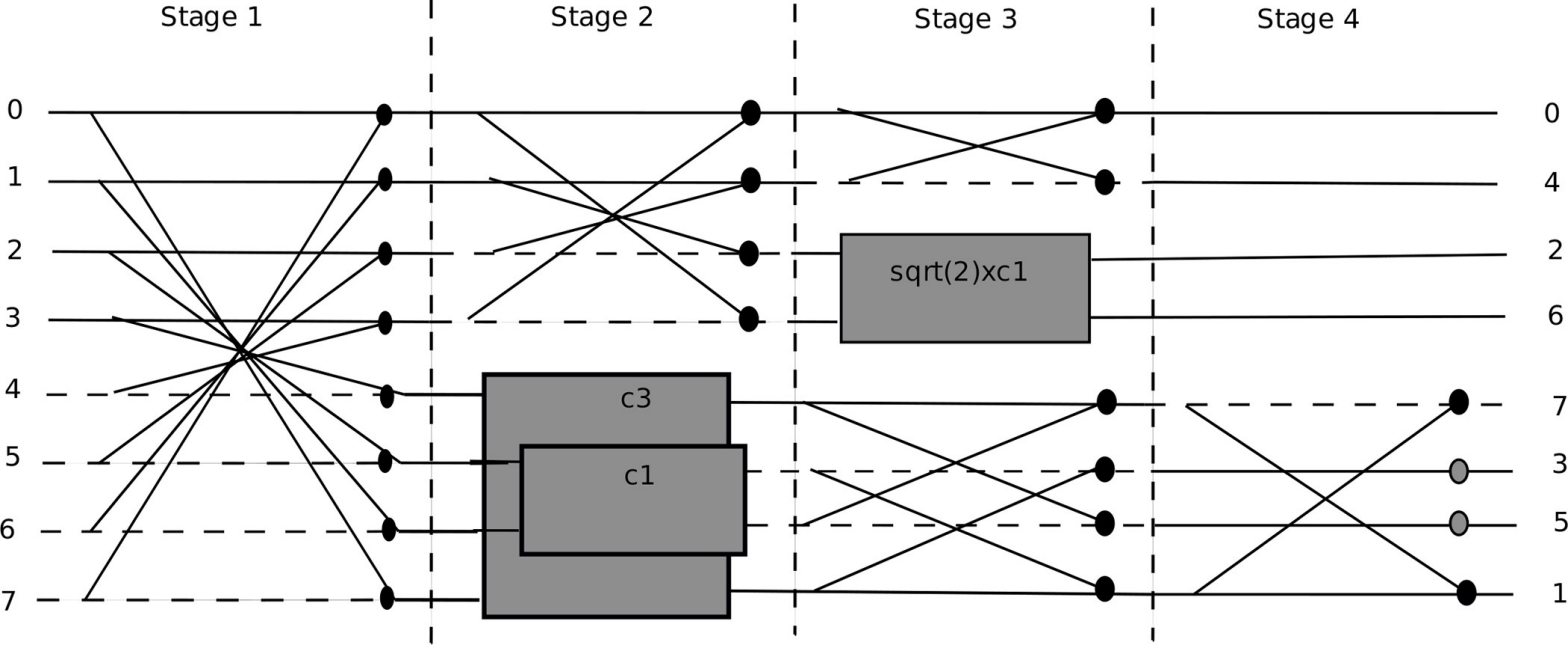
Block diagram of 8-point Loeffler 1D-DCT Algorithm.

**Fig 14:**
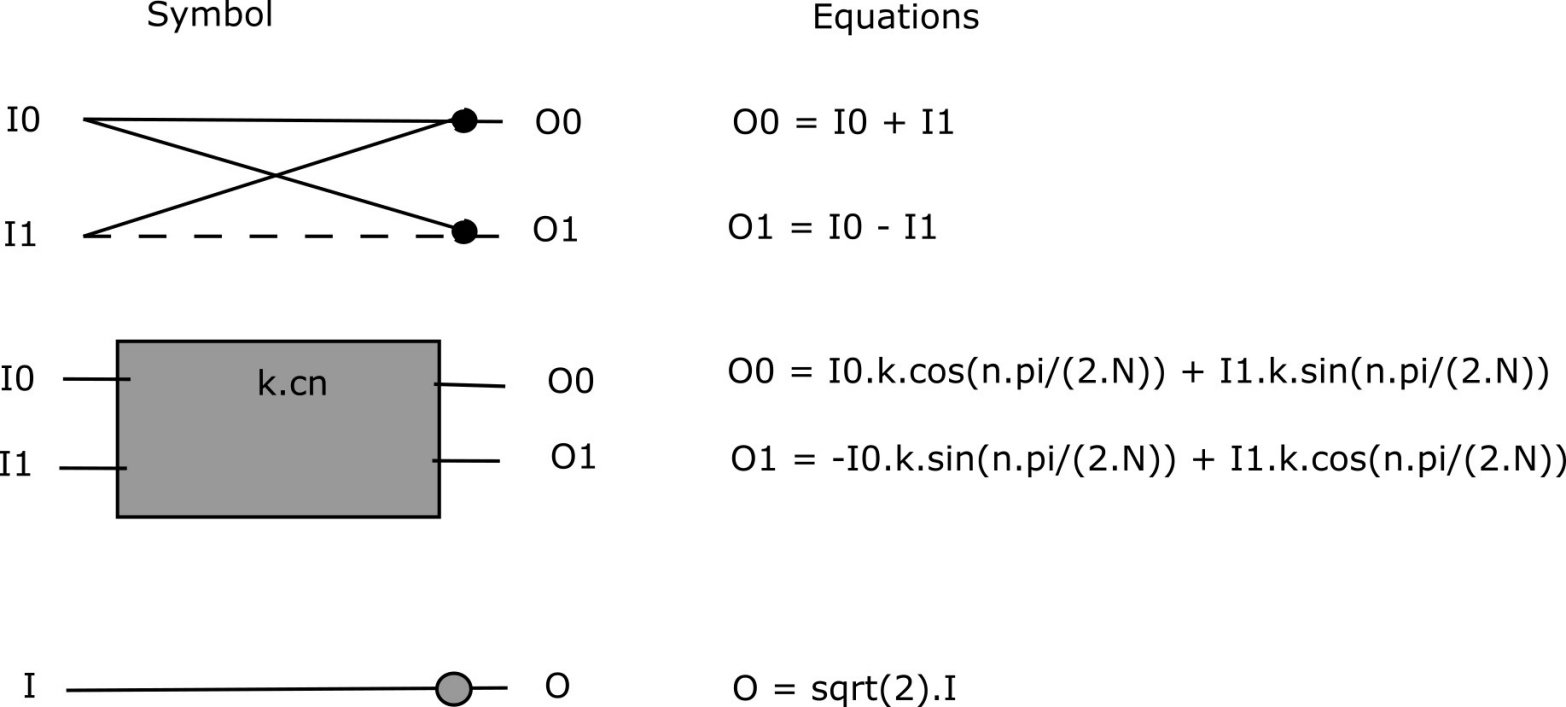
Symbols used for the Loeffler DCT algorithm.

When all threads are finished computing 1D-DCT values, then each of threads computes 1D-DCT values from 1D-DCT values already computed, along the columns, to yield 2D-DCT. Here each thread computes eight multiplications and eight additions for a (8×8) block. Figs 13 and 14 show block diagram of the Loeffler 1D-DCT algorithm [10], which has four stages and total 11 multiplications and 29 additions for 8-point 1D-DCT. Though four stages are in a sequential manner, each stage can be parallelized. Fig 12 shows block diagram for Algorithm 19, explaining the parallelization achieved using GPU threads, for conventional 2D DCT process.

Algorithms 20 & 21 show pseudocodes for the multithreaded Loeffler 1D-DCT algorithm. A (*col*×*row*) block of local threads is executing instructions in parallel, where *col* is 4 and *row* is 8. The difference image block is read into shared memory in parallel in a coalesced manner from global memory by local threads. Similarly, eight relevant DCT coefficients according to Figs 13 and 14 are read into shared memory.

**Algorithm 20:** Pseudocode for multithreaded (parallelized) Loeffler 1D-DCT algorithm.

 • 2D block (*col*×*row*) of local threads is executing the following instructions simultaneously.

 • Read corresponding difference macroblock in a shared memory in parallel from global memory.

 • Read *eight DCT* coefficients into shared memory.

**// stage 1**

   s[y][th_ind]=diff[y][th_ind]+diff[y][7−th_ind]

               s[y][7−th_ind]=diff[y][th_ind]–diff[y][7−th_ind]

 • Synchronize threads.

**// stage 2**

  s1[y][th_ind]=s[y][th_ind]+s[y][3−th_ind]

  s1[y][3−th_ind]=s[y][th_ind]–s[y][3−th_ind]

  m1[y][th_ind]=s[y][th_ind+4]*c[th_ind]

  m1[y][th_ind+4]=s[y][th_ind+4]*c[th_ind+4]

  *Synchronize threads*

  s1[y][2*th_ind+4]=m1[y][th_ind]+m1[y][3−th_ind]

  s1[y][2*th_ind+5]=m1[y][th_ind+4]+m1[y][7−th_ind]

 •Synchronize threads.

**Algorithm 20 (Continued):** Pseudocode (continued) for multithreaded Loeffler 1D-DCT algorithm.

**// stage 3**

  s2[y][0]=s1[y][0]+s1[y][1]

  s2[y][1]=s1[y][0]–s1[y][1]

  m1[y][2*th_ind]=s1[y][th_ind+2]*c[4*th_ind+1]

  m1[y][2*th_ind+1]=s1[y][th_ind+2]*c[4*th_ind+2]

  *Synchronize threads*.

  s2[y][th_ind+2]=m1[y][th_ind]+m1[y][th_ind+2]

  s2[y][3*th_ind+4]=s1[y][th_ind+4]+s1[y][th_ind+6]

  s2[y][th_ind+5]=s1[y][3*th_ind+4]–s1[y][6−th_ind]

  *Synchronize threads*.

**// stage 4**

  s3[y][th_ind]=s2[y][th_ind]

  s3[y][2*th_ind+4]=s2[y][2*th_ind+1]

  s3[y][7]=s2[y][7]–s2[y][4]

  s3[y][1]=s2[y][7]+s2[y][4]

  s3[y][th_ind+5]=sqrt(2)*s2[y][2*th_ind+3]

**Algorithm 21:** Pseudocode for the parallelization of 2D-DCT using Loeffler DCT algorithm.

 • 2D grid (2×*MB*_*X*×2×*MB*_*Y*) of global threads is executing the following instructions simultaneously.

 • 2D block (4 x row) of local threads is executing the following instructions simultaneously.

 • Read corresponding difference macroblock in a shared memory in parallel.

 • Synchronize threads.

 • The 1D-DCT values are computed by four threads (block rows) using Loeffler DCT

 • Synchronize threads.

 • The 1D-DCT values are computed by four threads (1D-DCT columns) using Loeffler DCT.

Among the eight DCT coefficients are given as

{$cos\frac{n\pi }{2N},\frac{n\pi }{2N}$, $-sin\frac{n\pi }{2N},cos\frac{n\pi }{2N}$}, for n equal to 3 and 1 and N equal to 8.

It can be noted that the last DCT coefficient is repeated. The reason is that it made multithreading coding efficient. The *th_ind* is the *x*-coordinate of the 2D local threads, which varies from 0 to 3. Similarly, *y* is *y*-coordinate of local threads varying from 0 to 7. The *diff* is difference image block. From stage 1 of Fig 13, the top four rows of output of stage 1 can be computed in parallel using four threads per block row. Similar is the case for bottom four rows. This is done in stage 1 of pseudocode 20 in Algorithm 20. Stage 1 has two arithmetic instructions per thread.

The result of stage 1 is stored in the shared memory array variable (*s*) as shown in pseudocode 20 in Algorithm 20. The eight additions (subtractions) of stage 1 of Fig 2 are reduced to two additions (subtractions) per thread per block row. All threads are synchronized before moving to stage 2. As can be seen from Fig 13, the top two rows of output of stage 2 can be computed in parallel. Similarly, the case is for third and fourth rows. This is shown by the first two instructions of stage 2 of the pseudocode 20 in Algorithm 20. These two instructions are executed by two threads, the *th_ind* equal to 0 and t*h_ind* equal to 1, per block row.

From stage 2 of Fig 13, the next four rows involve eight multiplications with DCT coefficients and four additions (subtractions). The eight multiplications are executed by four threads, i.e., two multiplications per thread per block row. This is shown by third and fourth instructions of stage 2 of pseudocode 20 in Algorithm 20. Following that, threads are synchronized. Then, these multiplications are added together by using two threads, *th_ind* equal to 0 and *th_ind* equal to 1. This is shown by sixth and seventh instructions of stage 2 of pseudocode 20 in Algorithm 20. The eight multiplications and eight additions (subtractions) of stage 2 of Fig 13 are reduced to two multiplications and four additions (subtractions) per thread per block row.

The result of stage 2 is stored in shared memory array variable (*s*1) as shown in pseudocode 20 in Algorithm 20, where *c* is the shared memory array variable holding required DCT coefficients. All threads are synchronized before moving to stage 3. The first two rows of stage 3 in Fig 13 have one addition and one subtraction and cannot be parallelized, so these are executed by a single thread as shown by the first two instructions of stage 3 of pseudocode 20 in Algorithm 20 (Continued). Rows 3 and 4 of stage 3 in Fig 13 have four multiplications and two additions (subtractions). The four multiplications are executed by two threads in parallel, *th_ind* equal to 0 and *th_ind* equal to 1. This is shown by third and fourth instructions of the pseudocode 20 in Algorithm 20 (Continued). The threads are synchronized, and multiplications are added again by two threads in parallel, *th_ind* equal to 0 and *th_ind* equal to 1, as shown by the sixth instruction of the pseudocode 20 in Algorithm 20 (Continued). The last four rows of stage 3 of Fig 13 have four additions (subtractions) that can be parallelized by two threads, *th_ind* equal to 0 and *th_ind* equal to 1. This is shown by seventh and eighth instructions of the pseudocode 20 in Algorithm 20 (Continued). The four multiplications and eight additions (subtractions) of stage 3 in Fig 13 are reduced to two multiplications and five additions (subtractions). All the threads are then synchronized. The result of stage 3 is stored in shared memory variable (*s*2). The first four rows of stage 4 of Fig 13 are rearrangement operations and can be executed by four threads, with two threads per assignment. The first and third assignments (first and third rows) in stage 4 of Fig 13 can be executed by two threads, *th_ind* equal to 0 and *th_ind* equal to 2. This is shown in pseudocode 20 in Algorithm 20 (Continued) by first instruction of stage 4. Similarly, the second and fourth assignments (second and fourth rows) of Fig 13 can be executed by two threads, *th_ind* equal to 0 and *th_ind* equal to 1. This is shown by the second instruction in stage 4 of pseudocode 20 in Algorithm 20 (Continued). Fifth and eighth rows of stage 4 of Fig 13 cannot be executed in parallel as they have different operations so are executed by a single thread as shown by third and fourth instructions of pseudocode 20 in Algorithm 20 (Continued).

However, the sixth and seventh rows of stage 4 of Fig 13 which involves two multiplications can be executed by two threads, *th*_*ind* equal to 0 and *th*_*ind* equal to 1. This is shown by the last instruction of pseudocode 20 in Algorithm 20 (Continued). The eight operations of stage 4 of Fig 13 are reduced to five operations. It is important to mention here that not all instructions of the multithreaded (parallelized) Loeffler DCT are executed by four threads. Some instructions are executed by two threads and some by a single thread. So, some instructions are conditional, however the conditional instructions are not shown in the pseudocode 20 of Algorithm 20 and Algorithm 20 (Continued). Overall, the maximum instructions per thread are 20, out of which there are five multiplications, 13 additions (subtractions) and 2 assignments. Algorithm 21 shows pseudocode of 2D-DCT using multithreaded Loeffler DCT with (4×8) threads per block.

If the multithreaded 2D-DCT from Algorithm 19 is compared with multithreaded 2D-DCT of Algorithm 21, in former case each thread is computing a single DCT coefficient with sixteen operations, in case of 1D-DCT, out of which there are eight multiplications and eight additions. However, it can be seen from Algorithm 17 that there is an inherent for-loop (inner most) which is being executed by each thread. Hence, there are more instructions for condition checking, iteration variable increment and the *goto* statement. So, there are total of 48 instructions per DCT coefficient per thread excluding synchronizing instructions and memory access instructions.

**Fig 15:**
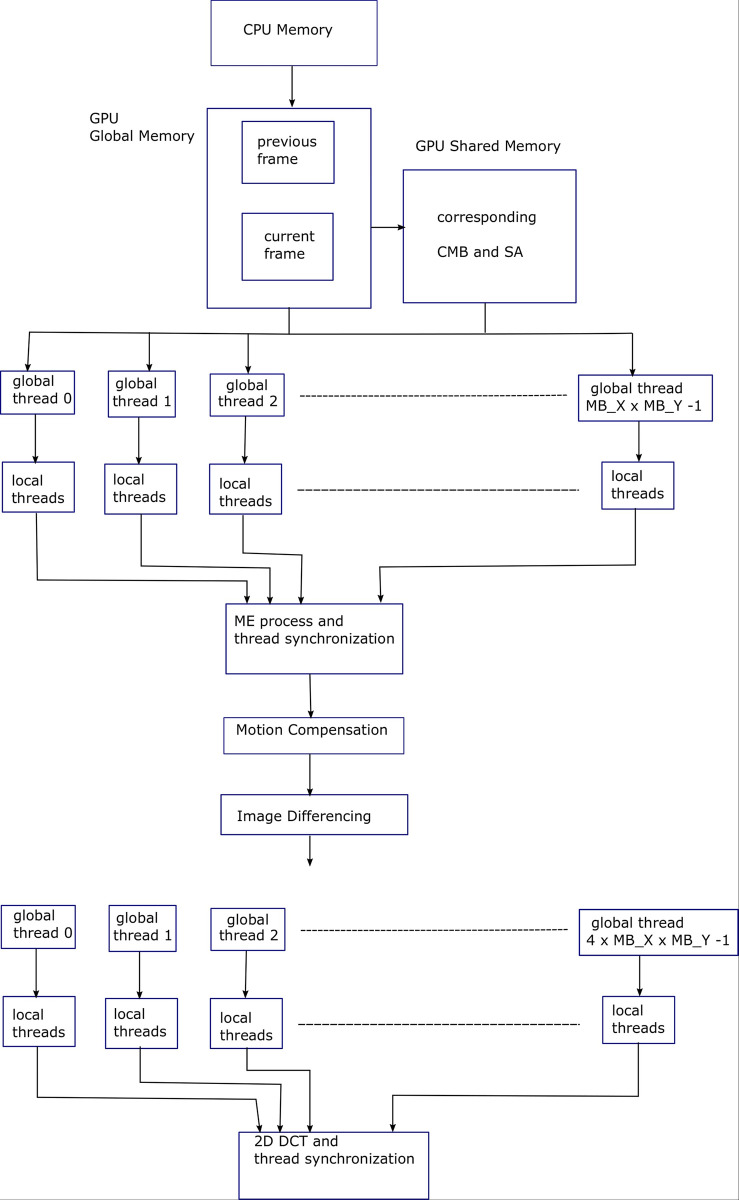
Block diagram for overall ME, motion compensation, image differencing and 2D DCT processes explaining the parallelization achieved using GPU threads.

Also, 2D-DCT per block is being computed by 64 threads, (8×8) in parallel, so there can be lot of shared memory access contentions. However, multithreaded Loeffler DCT is unrolled and has total of 20 instructions per 1D-DCT executed by each of four threads per block row, on average, excluding synchronizing instructions and memory access instructions. Also, 2D-DCT per block, in this case, is being executed by 32 threads, (4×8) in parallel, so there are comparatively less shared memory contentions. Because a multiplier has more delay as compared to an adder, multiplication operation can be a multicycle operation. Hence, reduced multiplication operations in the case of Loeffler DCT can lead to faster implementation. Since inverse 2D-DCT is also a part of video encoder and since it has almost same complexity as 2D-DCT, its implementation is not given here. Its parallelization follows the same steps as given above. Fig 15 shows block diagram for overall ME, motion compensation, image differencing and 2D DCT processes explaining the parallelization achieved using GPU threads.

### Improvement in parallelism from GPU hardware perspective

One difficulty that was noticed during the literature review was reduced throughput and large computational time of the implementations, making it difficult to achieve the real time throughput especially for UHD image sequences. There can be several reasons for this problem, e.g. too many off chip or global memory accesses, less utilization of SMs, GPU with low clock frequency, less level of parallelism, high computational complexity of algorithms, low occupancy of GPU, possibility of thread divergence etc. This work tries to mitigate these problems by using maximum and efficient parallelism, exploring all portions of the application, and ensuring efficient utilization of memory. However just parallelism alone could not assure the application’s real time response. Therefore, a low complexity ME algorithm and low complexity 2D DCT algorithms is suggested. This would serve to reduce the workload per thread, which together with the parallelism could meet real time constraint.

NVIDIA GeForce GTX 1080 GPU, which is based on Pascal architecture, has 20 SMs, each SM consists of 128 SPs that can run at a clock frequency of 1.6 GHz. The SPs are divided into four groups, each group consists of 32 SPs. Each group has associated 64 kB of register file (RF), i.e. 16384 x 32 bit, 8 load/store units, instruction buffer, warp scheduler and two dispatch units. In addition, the four groups share an L1 cache memory of 48 kB and a shared memory of 96 kB.

The input image has total number of 32400 (240x135) macroblocks of size 16x16 pixels each. According to Algorithm 2, the processing of all these macroblocks is assigned to a separate global or block thread. Inherently these thread blocks are assigned to the 20 SMs, i.e. each SM get assigned 1620 (32400/20) thread blocks. This ensures SMs are busy all the time in a balanced manner for high throughput. Since now the number of warps per block is less than one, it greatly reduces the occupancy and hardware utilization of the GPU resources resulting in less performance.

Since the GPU architecture is based on SIMT model, the warp scheduler schedules the same instruction to each SP, i.e. multiple threads are executing the same instruction. In this case there can be four thread blocks residing in each SM, using the corresponding SPs simultaneously. When the present thread blocks finish processing, the SM gives way to other thread blocks.

Since the data has spatial correlation among the adjacent macroblocks, so there is a lots of data reuse. In order to effectively exploit the data reuse, shared memory has been utilized in Algorithm 3. Shared memory is considerably faster than the global main memory. The computational time has been reduced significantly as mentioned in Table 1, since now most of the data accesses are from shared memory.

**Table 1:** Time consumption of the implementations.

Implementation		Time consumption (s)
**1**	*FS*_*Serial*	874
**2**	*FS*_*MB*_*par*_*direct*	11.4
**3**	*FS*_*MB*_*par*_*shared*_*mem*	5.9
**4**	*FS*_*MB*_*SA*_*par*	1.4
**5**	*EHDS*_*Serial*	16
**6**	*EHDS*_*MB*_*par*	0.24
**7**	*EHDS*_*MB*_*SAD*_*par*	0.15
**8**	*TZS*_*Serial*	69
**9**	*TZS*_*MB*_*par*	1
**10**	2*D*_*DCT*_*Serial*_*conventional*	9
**11**	2*D DCT Serial_Loef fler*	3
**12**	2*D*_*DCT*_*par*	0.1
**13**	*FS*_*MB*_*SA*_2*D*_*DCT*_*par*	1.54
**14**	*EHDS*_*MB*_*SAD*_2*D*_*DCT*_*par*	0.25

In order to further enhance the performance, Algorithm 4 utilizes search area parallelization. Corresponding to a search area of 32 x 32, the number of thread blocks remain the same but now there are 256 local threads, i.e. 16x16 CMVs, corresponding to each global thread block. The corresponding warp scheduler divides the local threads into warps, i.e. groups of 32 threads, so that there are total of 8 warps per thread block. Now there can be four warps residing in each SM, using the corresponding SPs simultaneously. During shared memory stalls, there is a fast context switching and the SM saves the data of the current threads into RF and gives way to other waiting warps depending upon the available resources. As RF is quite large, so multiple warps can reside in SM leading to enhanced parallelism. In this way multiple warps can be residing inside the corresponding SMs. As shown in Table 1, the computational time has been significantly reduced further as a majority of data accesses are from the shared memory and the global memory is accessed in coalesced manner. This means that multiple consecutive local threads are accessing contiguous locations of global memory to write into the shared memory. In this case, the search area and current macroblock are residing in the corresponding shared memory.

Yet another level of parallelism has been introduced by this work, i.e. SAD level parallelism, as expressed in Algorithm 7. For a 16 x 16-pixel sized macroblock, there are 16 local threads, each computing the corresponding 16 sum of absolute differences. There is now half warp per thread block per SM. Though thread occupancy has been reduced, the performance benefit came from fast shared memory accesses. As before, the search area and current macroblock have been written to shared memory and the global memory is accessed in a coalesced manner. The threads are synchronized using barrier instruction before adding the intermediate sums by using a single thread. In this case the search area is accessed in sequential manner. It needs to be mentioned here that the number of thread blocks remain the same as before.

A further level of parallelism has been introduced at the frame level, where multiple frames are being loaded to the GPU memory for the parallelization of the ME process, as mentioned in Algorithm 5. In the same way reconstruction process and image differencing has been parallelized as mentioned in Algorithms 13 and 15. While we observed significant benefits of the immense parallelization this research was able to incorporate, we found that the performance of the parallelization can be enhanced further by incorporating complexity reduction from the parallelization of fast EHDS algorithm that was recently proposed by the authors. The EHDS algorithm is faster and efficient than the TZS ME algorithm of HEVC standard, as mentioned in Algorithms 9 and 11, which utilizes multiresolution images and subsampled ME algorithm. Utilizing search area parallelism did not give any significant benefit as the search area has already been subsampled and there are very few CMVs available. However SAD level parallelism gave significant advantage as mentioned in Table 1. Again the number of thread blocks are same as before, however, each thread block now has 16 local threads for the computation of SAD in parallel. In addition, formation of multiresolution images has also been parallelized as mentioned in Algorithm 9.

The second most computationally intensive part of HEVC encoder is 2D DCT. In the conventional algorithm, 2D DCT is executed using 1D DCT, horizontally and vertically along the 8x8 block, using separable property of 2D DCT. One way of parallelizing 2D DCT is to allocate one local thread per DCT coefficient that includes 8 multiply accumulate operations per coefficient for 1D DCT. Intermediate results are saved in shared memory. This same process is repeated vertically on the saved results, resulting in 2D DCT, as mentioned in Algorithm 19. In this process there are 64 local threads operational per thread block. This means there are two warps operational per SM simultaneously. Since the DCT block size is now 8x8, so global thread blocks are increased by a factor of 4. In order to further enhance the performance of 2D DCT process, Loeffler’s fast DCT algorithm has been utilized, which has least number of multiplications and additions as mentioned in Fig 13. As mentioned in Algorithms 20 and 21, there is a block of local threads of size 4x8 per thread block. This means there is one warp per thread block per SM. Though occupancy has reduced, but in this case the workload per thread is much less as compared to the case for parallelization of conventional 2D DCT, leading to faster speed of the parallelized Algorithm 21. As mentioned in Table 1, the multithreaded Loeffler’s 2D DCT algorithm has higher performance than multithreaded conventional 2D DCT.

### Further possibility of parallelism and improvement and future directions

#### 1) Reducing shared memory accesses

Though shared memory is way faster than global memory of GPU, but it still effects performance if there are lot of shared memory accesses with contention. One way to alleviate the shared memory access load is to trade memory accesses with increased workload per thread. For example, in Algorithm 4, if instead of dedicating one local thread to the computation of one CMV SAD computation, one local thread is dedicated to the computation of 4 CMV SAD computations, the shared memory accesses can be saved as shown in the following algorithm 22,

**Algorithm 22:** Pseudocode for search area and macroblock level parallel FS ME algorithm with reduced shared memory accesses.

 ***for*** *i in* 1 *to frame_num loop*

  {

 • Load frames into GPU global memory.

 • 2D grid (*MB*_*X*×*MB*_*Y*) of global threads is executing following instructions simultaneously.

 • Load corresponding CMB and SA in the corresponding shared memories with 8 pixels per local thread for SA and two pixels per local thread for CMB.

 • 2D block $\left(\frac{SA\_X}{4}\times SA\_Y\right)$ of local threads executes the following instructions simultaneously.

 • Each local thread computes 8 SADs corresponding to 8 CMVs.

 • Synchronize threads.

 • The corresponding main global thread executes following instructions:

  ***for*** *l in* 1 *to SA_Y loop*

   ***for*** *m in* 1 *to SA_X loop*

 • Compute minimum SAD and the corresponding *MV*(*l*,*m*).

 }

In Algorithm 4, each local thread is executing the following assembly instructions during SAD computation,

ld reg1, CMB[k]

ld reg2, SA[l]

sub reg3, reg1, reg2

abs reg3, reg3

add reg4, reg4, reg3

ld reg1, CMB[k+1]

ld reg2, SA[l+1]

sub reg3, reg1, reg2

abs reg3, reg3

add reg4, reg4, reg3

————————

————————

where ld stands for load instruction, i.e. loading data from shared memory into RF, sub stands for subtraction operation, abs for absolute, and add for adding the intermediate result to the final SAD result. The instructions are shown for two absolute difference and addition operations. It can be seen that for one absolute difference (AD) operation, there are two shared memory accesses per local thread. This means for an SAD computation for 16x16 sized macroblock, there will be 512 shared memory accesses per local thread. It needs to be mentioned here that the syntax of the assembly code is not the actual code but a pseudocode that follows the following syntax.

Opcode [destination register], [source register 1], [source register 2]

On the other hand, according to Algorithm 22, the actual operations of local threads are as shown below in a pseudocode.

for l in 0 to 15 loop

for m in 0 to 15 loop

for i in 0 to 3 loop

SAD[i] = SAD[i] + abs(SA[th_y x 32 + th_x x 4 + 32 x m + l+ i]–CMB[m x 16 + l]

The above pseudocode is being executed by local threads of dimension 4 x 16. th_y is the vertical coordinate of the local thread block and th_x is the horizontal coordinate.

The corresponding optimized assembly code corresponding to the inner most *for* loop is as follows:

ld reg1, CMB[k]

ld reg2, SA[l]

sub reg3, reg1, reg2

abs reg3, reg3

add reg4, reg4, reg3

ld reg2, SA[l+1]

sub reg3, reg1, reg2

abs reg3, reg3

add reg4, reg4, reg3

ld reg2, SA[l+2]

sub reg3, reg1, reg2

abs reg3, reg3

add reg4, reg4, reg3

ld reg2, SA[l+3]

sub reg3, reg1, reg2

abs reg3, reg3

add reg4, reg4, reg3

————————

————————

As can be seen, that CMB pixel corresponding to the four CMV positions is accessed once and stored into the RF and reused among the computations. On the other hand, the computational load per local thread has increased. The shared memory accesses have been reduced from 4 x 512 = 2048 to 4 x 256 + 256 = 1280 and the computational load per local thread has been increased by a factor of 4. However, since each SP is dedicated to one thread, the computations on SP are much faster than shared memory accesses, which have stalls as well, compensating the increased load per thread leading to performance improvement.

#### 2) Using NVIDIA GPUs vector load store instructions

Using vector load store instructions, multiple contiguous data can be loaded from shared memory into RF and back with less instructions. However, vector arithmetic is not supported. Using vectorized load reduces number of instructions, reduces latency, and improves bandwidth utilization. The following pseudocode shows the vector operation.

int4 CMB1, SA1, SAD

for m in 0 to 15 loop

 for l in 0 to 3 loop

  {

   CMB1 = CMB[m x 16 + l x 3] // load four adjacent values from shared memory

   for i in 0 to 3 loop

    {

     SA1 = SA[th_y x 32 + th_x x 4 + 4 x i] //load four adjacent values from shared

     for j in 0 to 3 loop

      SAD[i] = SAD[i] + abs(SA1[j]–CMB1[j]

    }

  }

The above pseudocode is being executed by a group of local threads of dimension 16 x 4. The idea again is to reuse the data of CMB in the RF, without much accessing the shared memory. int4 is a vector data type, indicating array of four 32-bit integer values. In the above pseudocode, four values of CMB are loaded into CMB1 vector, once, and reused for the four CMV positions. Again the shared memory accesses have been reduced, while the work per thread has been increased.

It is important to mention here that there is a tradeoff between the number of local threads and the amount of data reuse for saving shared memory accesses. If threads are reduced to increase the data reuse, it saves shared memory accesses however it also increases work per thread.

#### 3) Using RF for data reuse

Another way for making the processing faster is to load blocks of data from shared memory into the RF and then reuse them. Since RF is faster than shared memory, it gives significant performance advantage. Similarly combining this technique with vector load store can further enhance the performance.

The above mentioned techniques are equally applicable to the processing of 2D DCT. Conventional 2D DCT involves matrix multiplication. So one row of matrix is being multiplies by multiple columns of the second matrix. Loading one matrix data from shared memory into RF and then reusing it can significantly improve the performance.

### Load balancing among threads

Motion Estimation is a highly regular and symmetric process. It involves dividing the image into equal sized MBs and then process them using same ME algorithm. This assures that the all the global thread blocks are receiving the same load. Similarly all the local threads are also receiving the same load. Similarly for the computation of 2D DCT, same work is assigned to all the threads, so there is a load balance among the threads.

## Results and discussion

The above-mentioned pseudocodes are implemented using the C and CUDA C languages [14]. The CPU utilized is the Intel(R) Core i7 running @ 2.9 GHz. The GPU utilized is the Nvidia GeForce GTX 1080. This GPU is based on the Pascal architecture, and it consists of twenty Pascal SMs and eight memory controllers. Each SM has 128 CUDA cores, 256 KB of register file, a 96 KB of shared memory unit and 48 KB of total L1 cache. Tied to each memory controller is 256 KB of L2 cache, i.e., total size of L2 cache in the GPU is 2048 KB. The base clock frequency is 1.6 GHz, i.e., the frequency at which CUDA cores are running.

The image sequence utilized in this study is the “park joy” with resolution (3840×2160) pixels [29]. The size of macroblock is (16×16). Thus, there are 3840/16, i.e., 240 macroblocks horizontally and 2160/16, i.e., 135 macroblocks vertically. Size of search area is (31×31) pixels, which is consistent with the maximum number of threads in a block in CUDA.

Table 1 shows time consumption in seconds of different implementations as described above. The *FS*_*Serial* is serial implementation of FS or exhaustive search ME algorithm (Pseudocode 1, Algorithm 1 and Pseudocode 6, Algorithm 6) on CPU. For 25 frames, the implementation consumes 874 seconds. The *FS*_*MB*_*par*_*direct* is parallelized implementation of FS ME algorithm (Pseudocode 2, Algorithm 2), at macroblock level, on GPU, in which 2D grid of global threads of dimension (240×135) pixels is utilized. However shared memory is not used, instead only global memory access is utilized.

The corresponding time consumption is 11.4 seconds. The speedup achieved is 874/11.4, which is 77. The speedup is defined as

$$SU=\frac{T_{CPU}}{T_{GPU}},$$ (7)

where, *T*_*CPU*_ is the time consumption of the CPU’s implementation and *T*_*GPU*_ is time consumption of GPU’s implementation.

The *FS*_*MB*_*par*_*shared*_*mem* is parallelized implementation of FS ME algorithm (Pseudocode 3, Algorithm 3), like implementation 2 above, however shared memory is utilized for fast and efficient data reuse by first loading search area and CMB in it. The time consumption is 5.9 seconds, and the corresponding speedup is 149. As can be seen the speedup is increased by making use of shared memory, which is the fastest memory after register file. The *FS*_*MB*_*SA*_*par* is the parallelized implementation of FS ME algorithm at macroblock and search area level (Pseudocode 4, Algorithm 4).

The time consumption is 1.4 seconds, and the corresponding speedup is 625. It is important to mention and note that SAD level parallelization (Pseudocode 7, Algorithm 7) cannot be implemented along with search area level parallelization. Since search area dimensions are greater than dimensions of a macroblock here, i.e., using search area parallelization is more beneficial than utilizing SAD level parallelization. Also frame level parallelization (Pseudocode 5, Algorithm 5) along with macroblock and search area level parallelization did not show significant reduction in computational time. The reason is that use of too many threads blocks serializes the implementation since there are fixed number of SMs, i.e., incorporating more threads after certain number of threads does not show significant benefit.

Though a significant speedup is achieved in FS ME algorithm, it is still not enough for a real time implementation. Thus, EHDS algorithm is utilized which has almost the same PSNR or bit rate distortion performance as that of FS ME algorithm, but with much reduced computational complexity. The *EHDS*_*Serial* is the serial implementation of EHDS algorithm (Pseudocode 8, Algorithm 8 and Pseudocode 10, Algorithm 10 and Pseudocode 6, Algorithm 6). As can be seen the time consumption is 16 seconds on CPU. The *EHDS*_*MB*_*par* is the parallelized implementation of EHDS ME algorithm at macroblock level (Pseudocode 11, Algorithm 11 and Pseudocode 9, Algorithm 9).

The time consumption is 0.24 seconds and the corresponding speedup relevant to EHDS_Serial is 67, whereas relevant to the *FS*_*Serial* it is 3642. The *EHDS*_*MB*_*SAD*_*par* is the parallelized implementation of EHDS ME algorithm at macroblock and SAD level (Pseudocode 11, Algorithm 11 and Pseudocode 9, Algorithm 9 and Pseudocode 7, Algorithm 7). Here global threads are parallelizing the macroblock level loops and local threads are parallelizing the SAD loop. As can be seen the time consumption is reduced to 0.15 seconds and the corresponding speedup relevant to *EHDS_Serial* is 107.

Here, search area level parallelization is not utilized as it is not very beneficial, because the search area has distant candidate motion vectors leading to non-coalesced memory access. Also, part of the candidate motion vectors is nondeterministic as is according to the EHDS algorithm. Since search area is serialized so it is now possible to exploit the SAD level parallelization, which is the case above.

For comparison purposes, authors implemented the TZS ME algorithm and parallelized it at macroblock level. As discussed in previous section, the algorithm has inherent dependency which hinders effective parallelization. Even if assumptions are made about predictive motion vectors, i.e., using temporal motion vectors (motion vectors of earlier frames) instead of using spatial motion vectors (spatially adjacent motion vectors in the same frame), the algorithm still has enough computational complexity making it not much suitable for real-time implementation of an encoder. As can be seen time consumption of parallelized implementation, *TZS*_*MB*_*par*, at macroblock level is one second, whereas time consumption of the *TZS*_*Serial* implementation, i.e., TZS on CPU, is 69 seconds. The speedup in this case is 69.

The 2*D*_*DCT*_*Serial*_*conventional* is serial implementation of the conventional 2D-DCT algorithm implemented as transform of transform (Pseudocode 16, Algorithm 16 and Pseudocode 17, Algorithm 17 and Pseudocode 18, Algorithm 18). The time consumption corresponding to 25 frames is 9 seconds. Similarly, 2*D*_*DCT*_*Serial*_*Loef fler* is the serial implementation of 2D-DCT in which Loeffler algorithm for 1D-DCT is used. The achieved time consumption is 3 seconds. The 2*D*_*DCT*_*par* is parallelized implementation and version of 2*D*_*DCT*_*Serial*_*Loef fler*. Its implementation is described in Pseudocode 21, Algorithm 21. As can be seen the efficient usage of shared memory and global and local threads, the time consumption has been reduced to 0.1 seconds. Pseudocode 19 in Algorithm 19 shows parallel 2D-DCT without Loeffler algorithm and is slightly inferior to that with the Loeffler algorithm.

The *FS*_*MB*_*SA*_2*D*_*DCT*_*par* is parallelized implementation of the parts of encoding process involving ME, reconstruction (motion compensation) (Pseudocode 13, Algorithm 13), image differencing (Pseudocode 15, Algorithm 15) and 2D-DCT. As can be seen the time consumption is 1.54 seconds. It also should be noted that the computational time of the reconstruction and image differencing has not been shown in Table 1, because the serial implementation of reconstruction process (Pseudocode 12, Algorithm 12) and the image differencing process (Pseudocode 14, Algorithm 14) has time consumption of one second for each process.

The parallelization of these processes leads to highly reduced and negligible time consumption. In addition, the inverse 2D-DCT is also a part of the encoding process and due to having symmetry with 2D-DCT, the algorithm has same computational time as 0.1 second which can be added in the overall time reduction. As can be seen with FS ME algorithm, the real-time constraint could not be achieved fully for 25 frames per second. However, for the *EHDS_MB_SAD_2D_DCT_par*, which is parallelized implementation of the parts of encoding process involving ME, reconstruction (motion compensation), image differencing and 2D-DCT; it can be seen that the encoding process has a time consumption of 0.25 seconds. The speedup achieved with respect to the serial implementations of the processes involving EHDS ME algorithm is 76.

The speedup of *EHDS_MB_SAD_par* with respect to serial implementation of FS ME algorithm, is 874/0.15, which is 5826.6. It can be concluded that there is enough time margin to cover the execution of remaining parts of encoder, i.e., quantization, inverse quantization and CABAC, in real time. It is also important to mention that flow control instructions such as the *if*, *switch*, *do*, *for*, and *while* can significantly reduce performance of a GPU since such instructions lead to thread divergence and the execution will have to be serialized [21]. Since CABAC algorithm is quite complicated with many flow control instructions, it would be better to implement it on CPU.

### Comparison with the state of the art

Table 2 shows comparison of the proposed implementation with the state-of-the-art, for the ME implementation. Since different ME algorithms and different CPU and GPU devices have been utilized by the state of the art, so in order to do a fair comparison of the state of the art with the proposed work, serial implementation of FS ME algorithm is taken as reference ME algorithm. Also the CPU and GPU utilized in the proposed work are taken as reference and the performance metrics of the state of the art are scaled accordingly.

**Table 2:** Comparison with the state of the art for ME.

	Scaled Speed up	Resolution	ME method	Scaled Throughput (frames/ second)
[16]	253.5	(1920×1080) pixels	FS ME	-
[17]	4275	(2560×1600) pixels	Fast ME	-
[18]	-	(1920×1080) pixels	Fast ME	22.8
[19]	-	(720×576) pixels	Improved FS ME	85
[20]	200	(2560×1600) pixels	Fast ME	-
[21]	-	(1280×720) pixels	Fast ME	65.8
[22]	432	(1920×1080) pixels	Fast ME	-
[25]	874	(3840×2160) pixels	TZS	25
**Proposed**	5826.6	(3840×2160) pixels	EHDS	> 150

In [16], Intel Core I7-3770 3.40 GHz CPU and a graphic card NVIDIA GeForce GTX480 is utilized. The peak performance of this GPU is 1.35 32-bit Tera floating point operations per second (TFLOPS). Modified FS ME algorithm is utilized and the GPU implementation is compared with the serial FS ME algorithm. The speedup, with respect to serial FS ME, reported is 50. The image resolution is 1920x1080 pixels. On the other hand the current work has utilized Intel(R) Core i7 CPU running at 2.9 GHz and GPU Nvidia GeForce GTX 1080 with peak performance of 8.8 TFLOPS. The image resolution utilized in the current work is 3840x2160 pixels. For comparison purpose, the scaled speedup of the work in [16] is calculated as,

Ratio of resolution = (3840x2160)/(1920x1080) = 4

Ratio of GPU peak performance = 8.8 TFLOPS/1.35 TFLOPS = 6.5

Ratio of CPU frequency = 3.4/2.9 = 1.17

Data transfer time overhead = 95/60 = 1.5

Scaled speed up = 50x4x6.5x1.17/(1.5 x4) = 253.5

Where the factor 95/60 tells about the data transfer time overhead from CPU to GPU as mentioned in [17].

In [17], Intel i7 2600 CPU with a base frequency of 3.4 GHz and GPU nvidia K40c with peak performance of 5.046 TFLOPS are utilized. The ME algorithm implemented is multilevel resolution motion estimation (MLRME for short) and the image resolution is 2560x1600. The speedup reported with respect to serial FS ME algorithm is 3150. The scaled speedup is then calculated as,

Ratio of resolution = (3840x2160)/(2560x1600) = 2

Data transfer time ratio = 95/60 = 1.5

Ratio of GPU peak performance = 8.8 TFLOPS/ 5.046 TFLOPS = 1.74

Ratio of CPU frequency = 3.4/2.9 = 1.17

Scaled speedup = 3150 x 1.17 x 1.74 x2 / (1.5x2) = 4275

In [18], the CPU used was Core 2 Quad E5607 clocked at 2.27GHz frequency and GPU is NVIDIA Tesla C2075 with peak performance of 1.028 TFLOPS. The throughput reported is 16 frames per second and image resolution is 1920x1080 pixels. The scaled throughput is computed as,

Ratio of resolution = (3840x2160)/(1920x1080) = 4

Ratio of GPU peak performance = 8.8 TFLOPS/ 1.028 TFLOPS = 8.56

Ratio of CPU frequency = 2.27/2.9 = 0.78

Data transfer time overhead = 95/60 = 1.5

Scaled throughput = 16x8.56 /(1.5x4) = 22.8 frames / sec

In [19], CPU is Intel (R) i5 450M 2.40GHz and GPU is NVIDIA Geforce 610M graphics card with peak performance of 0.0912 TFLOPS. The reported throughput is 26.45 frames per second and the image resolution is 720x576 pixels. The scaled throughput is computed as,

Ratio of resolution = (3840x2160)/(720x576) = 20

Ratio of GPU peak performance = 8.8 TFLOPS/ 0.0912 TFLOPS = 96.5

Ratio of CPU frequency = 2.4/2.9 = 0.82

Scaled throughput = 26.45 x 96.5 /(20x1.5) = 85 frames / sec

In [20], CPU frequency is 2.5 GHz and GPU is Tesla K40c with peak performance of 5.046 TFLOPS. Image resolution is 2560x1600 and the speedup with respect to FS ME is reported as 200. The scaled speedup is calculated as,

Ratio of resolution = (3840x2160)/(2560x1600) = 2

Ratio of GPU peak performance = 8.8 TFLOPS/ 5.046 TFLOPS = 1.74

Ratio of CPU frequency = 2.5/2.9 = 0.86

90/60 = 1.5

Scaled speedup = 200 x 0.86 x 1.74 x 2 /(2x1.5) = 200

In [21], CPU is Intel Core 2 Quad Q9400 2.66 GHz CPU and GPU is Nvidia GeForce 8800 GTX with peak performance of 0.345 TFLOPS. Image resolution is 1280x720 pixels and the GPU execution time for 60 frames is reported as 1688 ms. The scaled time and equivalent throughput is computed as,

Ratio of resolution = (3840x2160)/(1280x720) = 9

Ratio of GPU peak performance = 8.8 TFLOPS/ 0.345 TFLOPS = 25

Ratio of CPU frequency = 2.66/2.9 = 0.9

Data transfer time overhead = 95/60 = 1.5

Scaled time = 1688x9x1.5/25 = 911 ms

Scaled equivalent throughput = 60/911 ms = 65.8 frames/sec

In [22], CPU is Intel® Core™ i7 running at 2.80 GHz. GPU is Nvidia GTX480 with peak performance of 1.35 TFLOPS. Image resolution is 1280x720 and the reported speedup with respect to FS ME algorithm is 100. The scaled speedup is calculated as,

Ratio of resolution = (3840x2160)/(1280x720) = 9

Ratio of GPU peak performance = 8.8 TFLOPS/ 1.35 TFLOPS = 6.5

Ratio of CPU frequency = 2.8/2.9 = 1

Ratio of search range = (64x64)/(32x32) = 4

Data transfer time overhead = 95/60 = 1.5

Scaled speedup = 100 x 1 x 6.5 x 9x4 /(4x9x1.5) = 432

Since the search range in [22] is 64x64, whereas the search range in the current work is 32x32, so it is included in the above calculations.

For comparison purposes, HEVC TZS algorithm has also been implemented and it gives a speedup of 874/1 which is 874, as compared to FS ME algorithm.

As can be seen from Table 2, the proposed implementation has the highest throughput of greater than 150 frames per second and highest speedup of 5826, corresponding to image sequence resolution of (3840×2160) pixels. The symbol “-” in the last column of the table shows either throughput does not meet real-time constraint, or it is not mentioned. The PSNR (quality of reconstruction) obtained corresponding to FS ME, TZS ME, and EHDS algorithms are 25 dB, 24.83 dB, and 26 dB [6], respectively.

In addition another comparison has been made of the proposed encoder with the encoder implemented by Nvidia [26, 27]. From Table 1, time consumed by ME, Image differencing, 2D DCT and Reconstruction stages is 0.25 seconds for 25 frames of resolution 3840x2160 pixels. Since 2D DCT and 2D Inverse DCT are symmetrical, hence 2D Inverse DCT will approximately take 0.1 seconds. Similarly, total of 0.1 seconds were taken by Quantization and Inverse quantization stages, leading to total of 0.45 seconds excluding CABAC stage. The scaled time for Nvidia L40 GPU is calculated as,

Ratio of resolution = (7680x4320)/(3840x2160) = 4

Ratio of GPU peak performance = 90.5 TFLOPS/8.8 TFLOPS = 10.28

Data transfer time overhead = 95/60 = 1.5

Scaled time consumption = 0.45x4x1.5/10.28 = 0.26.

That is, the time consumed by the proposed encoder on Nvidia L40 GPU is approximately 0.26 seconds for 25 frames of resolution 7680x4320 pixels. Thus the proposed encoder can execute nearly execute 100 frames in one second. Since time consumption of CABAC stage is not included, hence it can be said that the proposed encoder has nearly same performance as Nvidia HEVC encoder.

## Conclusion

To boost performance of the contemporary video encoder (i.e., high efficiency video coding (HEVC) encoder), authors proposed an efficient real-time implementation of the ME and 2D-DCT structures using the Graphics processing unit, i.e., the NVIDIA GeForce GTX 1080. In addition, authors also implemented the reconstruction (i.e., ME compensation) and image differencing processes of the encoder for real-time applications. First, the FS algorithm which is thought exhaustive is the golden standard for ME process in terms of quality; so, it was considered for parallelization. Using global and local threads, tiling and shared memory reuse, the computational time is significantly reduced, but it did not meet real-time constraints. Next, authors considered a low complexity efficient ME algorithm known as EHDS ME algorithm for GPUs’ implementation. Unlike the TZS algorithm of HEVC, this algorithm does not have dependency on spatially adjacent motion vectors and so, it is best choice for efficient parallelization. Experimental results show that throughput of EHDS ME algorithm alone on GPUs corresponding to UHD sequences is greater than 150 frames per second. Then, authors implemented 2D-DCT structure on GPU. Two implementations are presented: i) first one is maximal multithreading of 2D-DCT and ii) second one is an efficient multithreaded (parallelized) 2D-DCT implementation based on the Loeffler’s DCT algorithm; the second implementation is found faster experimentally. The encoding process excluding CABAC stage take 0.45 seconds corresponding to 25 frames of the UHD sequences. Hence, this proposed encoder implementation using GPU can safely be applied in numerous real-time applications.
